# Production of superoxide from Photosystem II in a rice (*Oryza sativa* L.) mutant lacking PsbS

**DOI:** 10.1186/s12870-014-0242-2

**Published:** 2014-10-24

**Authors:** Ismayil S Zulfugarov, Altanzaya Tovuu, Young-Jae Eu, Bolormaa Dogsom, Roshan Sharma Poudyal, Krishna Nath, Michael Hall, Mainak Banerjee, Ung Chan Yoon, Yong-Hwan Moon, Gynheung An, Stefan Jansson, Choon-Hwan Lee

**Affiliations:** Department of Integrated Biological Science and Department of Molecular Biology, Pusan National University, Busan, 609-735 Korea; Umeå Plant Science Center, Department of Plant Physiology, Umeå University, Umeå, SE-901 87 Sweden; Crop Biotech Institute, Kyung Hee University, Yongin, 446-701 Korea; Department of Chemistry, Pusan National University, Jangjeon-dong, Keumjung-gu, Busan, 609-735 Korea; Department of Biology, North-Eastern Federal University, 58 Belinsky Str, Yakutsk, 677-027 Republic of Sakha (Yakutia) Russian Federation; Institute of Botany, Azerbaijan National Academy of Sciences, Patamdar Shosse 40, Baku, AZ 1073 Azerbaijan; Department of Biology, Mongolian State University of Agriculture, Zaisan, Ulaanbaatar, 17024 Mongolia

**Keywords:** Photoprotection, PsbS, ROS, Superoxide, Photosynthesis, NPQ, Rice

## Abstract

**Background:**

PsbS is a 22-kDa Photosystem (PS) II protein involved in non-photochemical quenching (NPQ) of chlorophyll fluorescence. Rice (*Oryza sativa* L.) has two *PsbS* genes, *PsbS1* and *PsbS2*. However, only inactivation of *PsbS1*, through a knockout (PsbS1-KO) or in RNAi transgenic plants, results in plants deficient in qE, the energy-dependent component of NPQ.

**Results:**

In studies presented here, under fluctuating high light, growth of young seedlings lacking PsbS is retarded, and PSII in detached leaves of the mutants is more sensitive to photoinhibitory illumination compared with the wild type. Using both histochemical and fluorescent probes, we determined the levels of reactive oxygen species, including singlet oxygen, superoxide, and hydrogen peroxide, in leaves and thylakoids. The PsbS-deficient plants generated more superoxide and hydrogen peroxide in their chloroplasts. PSII complexes isolated from them produced more superoxide compared with the wild type, and PSII-driven superoxide production was higher in the mutants. However, we could not observe such differences either in isolated PSI complexes or through PSI-driven electron transport. Time-course experiments using isolated thylakoids showed that superoxide production was the initial event, and that production of hydrogen peroxide proceeded from that.

**Conclusion:**

These results indicate that at least some of the photoprotection provided by PsbS and qE is mediated by preventing production of superoxide released from PSII under conditions of excess excitation energy.

**Electronic supplementary material:**

The online version of this article (doi:10.1186/s12870-014-0242-2) contains supplementary material, which is available to authorized users.

## Background

Light energy is converted to chemical energy during photosynthesis. However, because excess light is harmful, plants engage several protective mechanisms, including non-photochemical quenching (NPQ) of chlorophyll (Chl) fluorescence. NPQ is subdivided into three components that involve relaxation kinetics under darkness followed by a period of illumination. The first component, qE, relaxes quickly (within seconds to minutes) and is triggered by an increase in the trans-thylakoid proton gradient, or ΔpH. The second component, qT, relaxes more slowly and is a state transition phenomenon. The last component, qI, with the slowest relaxation, is a rather ill-defined component which traditionally includes a non-relaxing component related to irreversible damage, such as the inactivation of D1 protein in the Photosystem (PS) II reaction center [1,2]. Recently, the third very slow component, qZ was proposed, which depends on zeaxanthin [3]. Zeaxanthin directly or indirectly contributes to all NPQ mechanisms except qT [2].

The major component, qE, is dependent on three factors: the ΔpH [4], pigments in the xanthophyll cycle [5], and a 22-kDa PSII protein called PsbS [6]. These control qE in an integrated manner. Although the signal largely disappears when one factor is absent, qE can still be induced in the absence of PsbS, albeit much more slowly [7]. The qE signal is characterized by several activities, e.g., light-induced absorbance changes at 535 nm [8], shortening of a specific Chl fluorescence lifetime component from ~2.0 to ~0.4 ns [9], formation of carotenoid cation radicals [10], or changes in the configuration of neoxanthin molecules in the light-harvesting complex (LHC) II [11]. Alterations in absorbance and the Chl fluorescence lifetime often reflect structural changes in pigment-protein complexes of the thylakoid membranes.

The role of the PsbS protein in qE was first described in *npq4-1* mutants of *Arabidopsis thaliana* that lack *PsbS* [6]. Although this protein is evidently necessary for qE, *Arabidopsis* mutants completely lacking PsbS show normal photochemistry without any visible phenotype under controlled-environment conditions of non-fluctuating light [6,12]. However, when grown in the field or under rapidly fluctuating moderate light in a laboratory, those mutants produce fewer seeds than wild-type plants [13] and also show retarded growth [14]. The function of PsbS in qE development remains unclear, and the role of protonation of its glutamate residues in Chl fluorescence quenching is still debated [15,16]. Two thylakoid lumen-exposed glutamate residues of PsbS sense shifts in pH [17] and induce conformational changes that control qE [18]. PsbS does not seem to bind pigments [19] but may either interact with CP29 [20] or induce conformational modifications in it that modulate the energy of the Chl/zeaxanthin charge-transfer state [21]. Recent data have provided information on how PsbS controls the conformation and organization of PSII supercomplexes [22-25]. Recently have been shown that PsbS controls over photosynthesis in fluctuating light which optimize the photoprotective processes [26].

When NPQ is inhibited, one might expect more reactive oxygen species (ROS) to be produced in the chloroplasts. Powerful ROS include the highly reactive singlet oxygen [27], the superoxide anion radical, and hydrogen peroxide [28]. Biotic- and abiotic-stress conditions lead to an imbalance between ROS generation and scavenging; those accumulated ROS can cause damage to cells near the sites where they are generated [29]. Even though ROS are scavenged by diverse antioxidative defense substances (e.g., antioxidant enzymes and antioxidants such as ascorbate, tocopherol, and glutathione; [30,31]), ROS levels may rise rapidly following environmental changes [32]. Due to their highly reactive nature, ROS react with a wide range of molecules in biological organisms and can damage these molecules with consequences that may be fatal to the cell or even the plant [33].

The main source of ROS in chloroplasts is the electron transport chain; the generation site for each ROS depends upon the stress applied [30,34,35]. Singlet oxygen is a byproduct of photosynthesis, mainly formed at PSII [36] but also in other locations where triplet Chl molecules are produced. Generally, three different sites within the photosynthetic apparatus are associated with singlet oxygen production: i) the PSII reaction center; ii) the antennae of the LHC, and iii) the PSI acceptor site [37]. The destructive effect of singlet oxygen on D1 protein within the PSII reaction center is well understood [38,39]. However, little is known about how singlet oxygen influences other components of the thylakoid membrane. The singlet oxygen produced in *flu* mutants of *Arabidopsis* strongly affects ATP synthase activity and causes changes in NPQ, although its production site differs from those mentioned above [40]. The water–water cycle is considered the main source for superoxide production on the reducing side of PSI, helping plants to dissipate excess light energy by increasing the rate of electron transport and lowering the luminal pH [41-43]. Generation of superoxide within PSII has also been reported [44-46]. The next site for superoxide production is the plastoquinone pool [47]. There, superoxide is rapidly dismutated to the more stable hydrogen peroxide by superoxide dismutase (SOD) [28]. If superoxide is produced within the thylakoid membrane [48] where SOD is absent, hydrogen peroxide can be produced by the reduction of superoxide by plastohydroquinone PQH_2_ [47,49], and the same pathway also occurs within mitochondrial membrane [50].

To elucidate the role of PsbS protein in the photoprotective mechanism of NPQ, we investigated the consequences of eliminating this protein, especially on the generation of ROS. We found that superoxide produced at PSII was greater in PsbS-knockout rice leaves than in the wild type leaves. However, the levels of superoxide produced at PSI did not differ between the mutants and wild-type plants. Therefore, we suggest that PsbS protects against superoxide production at PSII when excess energy is absorbed by the PSII antennae.

## Results

### Isolation of a rice PsbS knockout plant and generation of PsbS RNAi transgenic plants

The rice genome has two *PsbS* genes -- *OsPsbS1* (LOC_Os01g64960) on Chromosome 1 and *OsPsbS2* (LOC_Os04g59440) on Chromosome 4 [51]. *OsPsbS1* and *OsPsbS2* encode proteins of 268 and 254 amino acids, respectively. *Arabidopsis* PsbS (AtPsbS) shares 68% sequence similarity with OsPsbS1 and 71% with OsPsbS2; the two rice proteins show 72% overall amino acid identity with each other.

**Figure 1:**
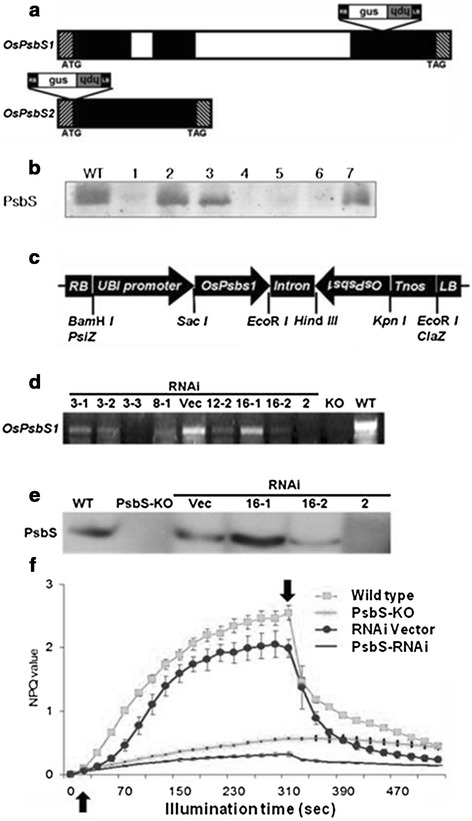
**Characterization of PsbS-KO and PsbS-RNAi rice plants. (a)** Schematic diagrams of the rice PsbS genes and the T-DNA insertion positions. The exons are shaded and introns are indicated with open boxes. For *OsPsbS1*, T-DNA was inserted into 3rd exon; for *OsPsbS2*, T-DNA was inserted into the beginning of the exon. **(b)** Western blot analysis of putative homozygous and heterozygous plants. PsbS protein was detected with a PsbS-specific polyclonal antibody. Lines 1 to 7 were segregated in the T2 generation of *OsPsbS1* plants. **(c)** Schematic diagram of rice PsbS-RNAi vector. RB, right border; UBI promoter, ubiquitin I promoter; OsPsbS1, inverted repeat of a unique 102-bp fragment of coding region for PsbS gene; Intron, 204-bp portion of the 3^rd^ intron of OsEMF1 gene (AF326768); Tnos, nopaline synthase terminator; LB, left border. **(d)** Transcript levels for PsbS gene in wild-type, PsbS-KO, PsbS-RNAi, and vector-only rice. Numbers indicate Lines of the PsbS-RNAi transformants. **(e)** Western blot analysis of PsbS-RNAi transformants and vector-only rice. Wild-type (WT) and PsbS-KO plants were used as positive and negative controls. **(f)** Light-induced NPQ generation in leaves (intensity of actinic light: 700 *μ*mol photons m^−2^ s ^−1^). Up arrow, light switched on; down arrow, light switched off. Each point represents mean of at least 4 experiments (SD indicated by bar). NPQ was calculated as described in Methods.

We selected PsbS-KO, a putative knockout mutant for *OsPsbS1*, from a pool of T-DNA insertion rice lines. It had been generated by transformation with a T-DNA vector (pGA2707) containing a promoterless *GUS* gene next to the left border (LB) of the T-DNA [52]. Sequencing via inverse-PCR of the region flanking that insertion [53] revealed that the T-DNA was inserted in the 3rd exon of *OsPsbS1* (Figure 1a). By genotyping multiple segregating lines in the T_2_ generation, we selected four plants homozygous for the T-DNA insertion. The result of the genotyping of PsbS1 line is shown in Additional file 1: Figure S1. Western blotting with a PsbS-specific antibody from AgriSera indicated that all four of the homozygous mutant plants lacked PsbS (Figure 1b). In those plants, the NPQ value that developed within 5 min was approximately 0.4 (Figure 1f), which is similar to that reported for the *Arabidopsis npq4-1* mutant [54]. A more detailed analysis of NPQ relaxation (or dark recovery of developed NPQ) indicated that qE was completely lacking in PsbS-KO leaves.

A KO mutant plant for *OsPsbS2* was also chosen from the pool of T-DNA insertion lines. Its flanking sequence revealed that the T-DNA was inserted in the sole exon near its start codon (Figure 1a). This rice gene product shares very high sequence similarity with AtPsbS, and exposure of etiolated seedlings to red and blue light induces a several-fold increase in the steady-state level of *OsPsbS2* transcripts [55]. Nevertheless, this rice mutant exhibited no visible phenotypic deviations with respect to the wild type, and their NPQ levels were also very similar. No *OsPsbS2* product was found in *OsPsbS1* knockout mutant plants when two different PsbS-antibodies were used, suggesting that such a product could not be detected by these antibodies. This was probably because either the immunological characteristics of OsPsbS and the *Arabidopsis* protein differ from that of the *OsPsbS2* gene product or else the protein encoded by *OsPsbS2* does not accumulate in the chloroplasts. These data are also consistent with previous genetic evidence that *OsPsbS1,* but not *OsPsbS2*, is co-localized with a QTL for NPQ [56]. Thus, our subsequent characterization focused solely on *OsPsbS1*, which we refer to as *OsPsbS* hereafter.

To confirm that the reduced NPQ of PsbS-KO leaves was caused by the insertion in *OsPsbS1* and not by either the insertion of multiple T-DNAs or other genetic differences, we used RNA interference (RNAi) technology to generate transgenic rice with significantly reduced PsbS protein levels. Plants were transformed with an RNAi construct that contained an inverted repeat of a unique 102-bp region of *OsPsbS1*, with a portion of the pBSIIKS vector serving as a linker and driven by the ubiquitin I promoter (Figure 1c). Transformants were screened by RT-PCR for *OsPsbS1* transcripts (Figure 1d) and confirmed by western blotting (Figure 1e). From these, we identified three RNAi lines with varying *OsPsbS1* transcript and PsbS protein levels. Among them, Line #2 produced little or no PsbS. Its NPQ development level (Figure 1f) and its corresponding light curve (Additional file 1: Figure S2) were comparable to those found from the PsbS-KO line. However, electron transport rates were similar between all investigated samples and their wild type counterparts (Additional file 1: Figure S3).

### Lack of PsbS protein in rice plants results in increased sensitivity to photoinhibitory illumination

**Figure 2:**
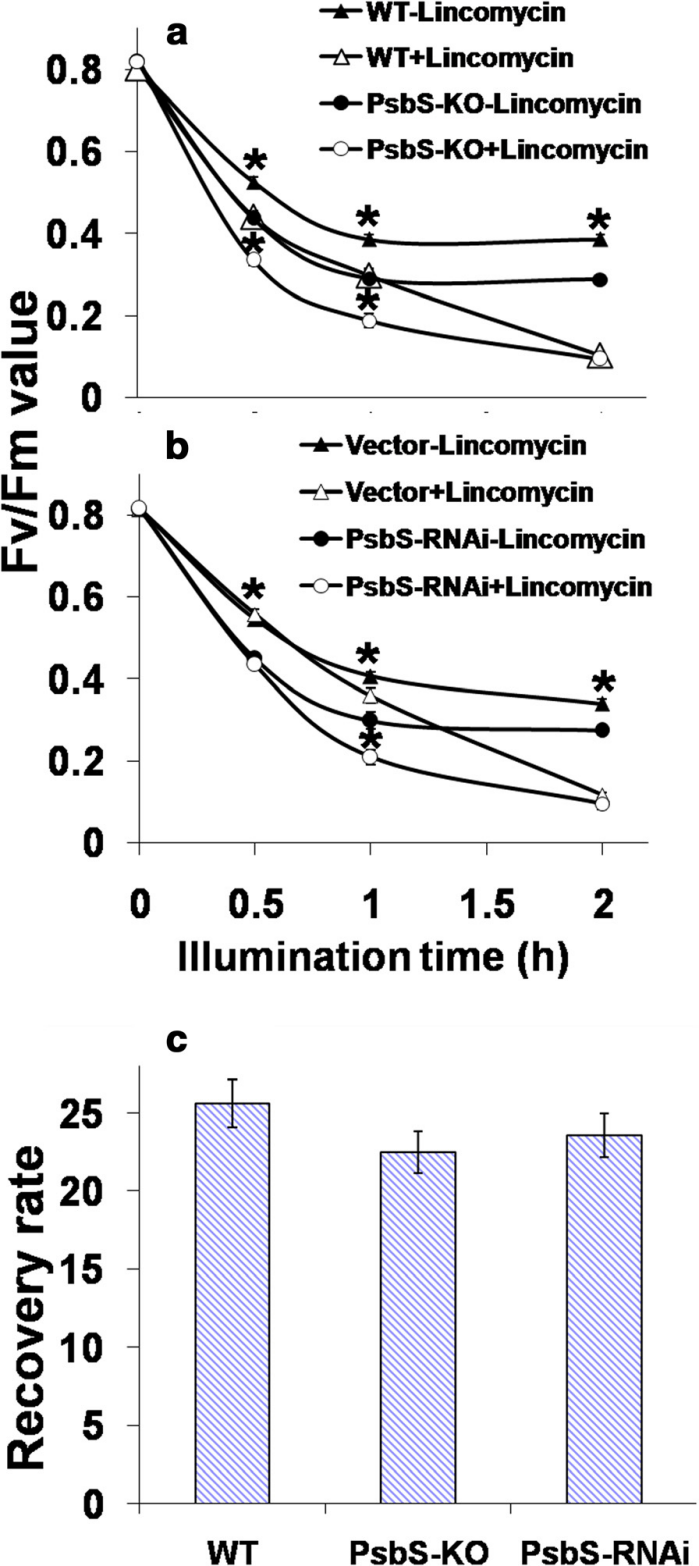
**Photoinhibition of PSII defined as decrease in Fv/Fm during photoinhibitory illumination. (a)** wild-type and PsbS-KO plants. **(b)** PsbS-RNAi and vector-only plants. Leaves were illuminated at 2,000 *μ*mol photons m^−2^ s^−1^ for photoinhibition in absence (closed symbols) or presence (open symbols) of 2 mM lincomycin. **(c)** Recovery of damaged PSII for 2 h in absence of lincomycin under dim light (50 *μ*mol photons m^−2^ s^−1^). Recovery rate was calculated as % increase in Fv/Fm after 2-h recovery period relative to decreased value before recovery began. Each point represents mean of at least 4 experiments (SE indicated by bar) and the asterisks denote the results that were significantly different from those in the wild type (*P < 0.05). The statistical significance was evaluated using the Student’s *t*-test.

At the whole-plant level, an *Arabidopsis* mutant (*npq4-1*) lacking the PSII protein PsbS has no visible phenotype except for reduced fitness when grown under either oscillating light or in the field [13,14]. We also observed that the growth rates of PsbS-KO and PsbS-RNAi rice plants under fluctuating light were significantly reduced (Additional file 1: Figure S4), and that grain yield from PsbS-KO plants was only about 30% of that reported from the wild type [57]. Under strong illumination (1,200 μmol photons m^−2^ s^−1^, white light), *npq4-1* becomes more susceptible to photoinhibition than wild-type plants [58]. When we exposed leaf segments to photoinhibitory illumination (2,000 μmol photons m^−2^ s^−1^ for 2 h), values calculated for Fv/Fm in the rice PsbS-KO mutant and RNAi plants dropped very rapidly, to about 40% of the level for dark-adapted controls (Figure 2a). By contrast, the Fv/Fm in the wild type was reduced to about 55% of the dark-adapted control. In all plant types, this decline was largely completed within 1 h of treatment, and no further decrease was observed thereafter (Figure 2a,b - closed symbols). The initial decline in Fv/Fm probably resulted because photodamage to PSII occurred more rapidly than it could be repaired. Moreover, the significant reduction in this rate of decline after 1 h was due to activation of the PSII recovery process [59,60]. The initial rates of photodamage were apparently higher in both PsbS-KO and RNAi leaves than in the wild type; however, recovery seemed to be activated in a similar manner regardless of genotype (Figure 2a,b).

Photoinhibition is a complex process entailing photodamage, repair of D1 protein of PSII, and re-assembly of active PSII [61]. Leaf infiltration with lincomycin, an inhibitor of protein synthesis in the chloroplasts, allows one to assess this process in isolation. Here, when lincomycin was applied, the rates of photodamage in both PsbS-KO and PsbS-RNAi leaves were higher than in the treated wild type. Blockage of the recovery process meant that the Fv/Fm for all three plant types continued to decrease until it reached ~10% of the dark-adapted value. This demonstrated that PsbS-KO and PsbS-RNAi leaves are more susceptible to PSII photodamage.

To monitor how leaf segments recovered in the absence of lincomycin after photoinhibition, we measured changes in Fv/Fm after 2 h of exposure to dim light (50 μmol photons m^−2^ s^−1^) at room temperature. The illumination used in this experiment resulted in ~40% and ~50% reductions in the Fv/Fm values of PsbS-KO and wild-type leaves, respectively. After a 2-h recovery period, Fv/Fm of the PsbS-KO, PsbS-RNAi, and wild type reached 82%, 84% and 90% of their dark-adapted values, respectively (Figure 2c). This indicated that the recovery process, including repair and re-assembly, is normal in PsbS-KO and PsbS-RNAi leaves.

### Superoxide and hydrogen peroxide production is higher in PsbS-deficient rice leaves

**Figure 3:**
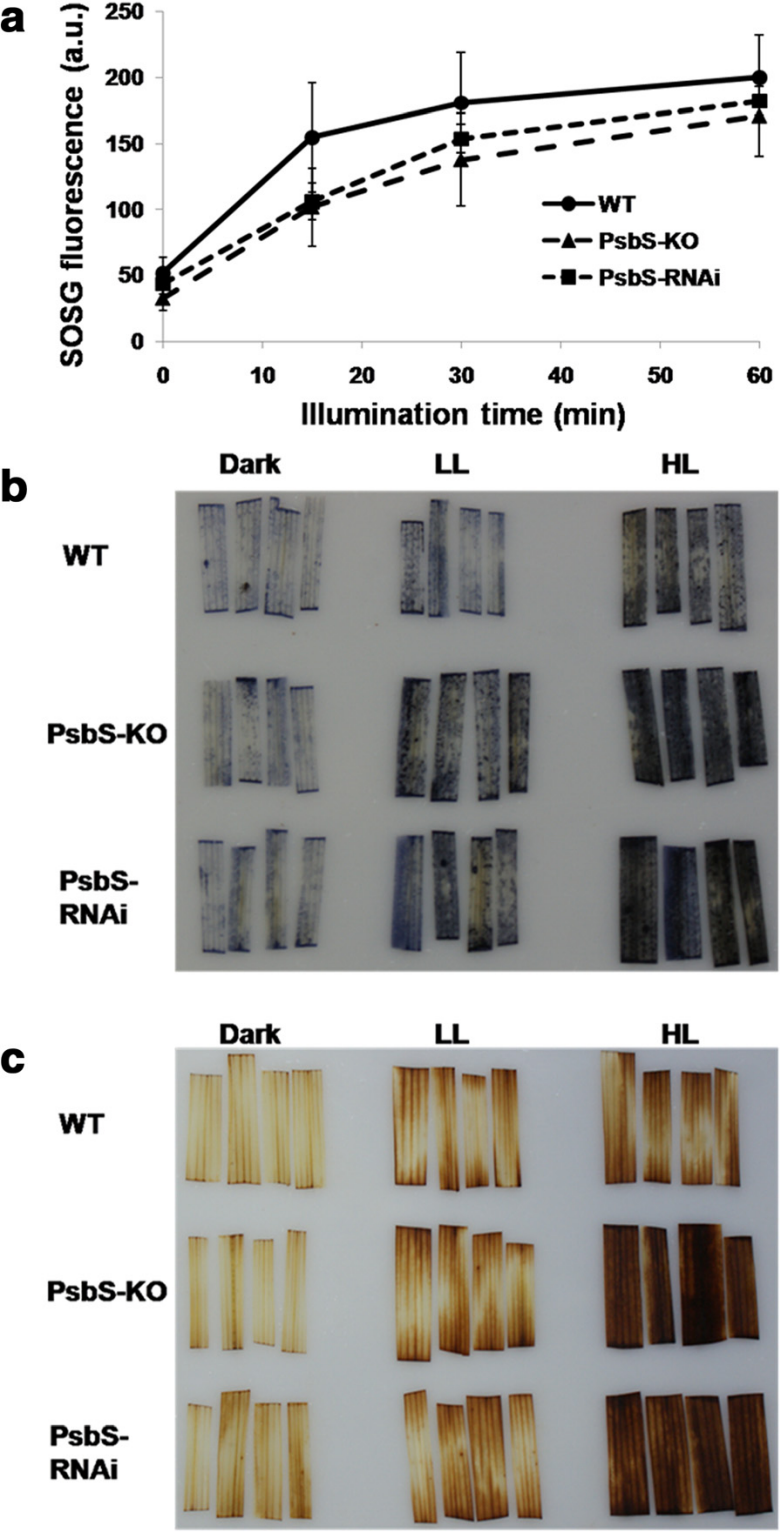
**ROS production in rice. (a)** Detection of singlet oxygen in leaves, as monitored by increase in SOSG fluorescence emission at 530 nm. Leaf segments were vacuum infiltrated with 200 *μ*M SOSG solution before being illuminated at 2,000 *μ*mol photons m^−2^ s^−1^. **(b)** Production of superoxide anion radicals. Histochemical staining with NBT in wild-type (WT), PsbS-KO and PsbS-RNAi leaves incubated under darkness for 2 h (Dark), under moderate light at 200 *μ*mol photons m^−2^ s^−1^ (LL), or under photoinhibitory illumination at 2,000 *μ*mol photons m^−2^ s^−1^ (HL). **(c)** Production of hydrogen peroxide. Histochemical staining with DAB in wild-type (WT), PsbS-KO and PsbS-RNAi leaves under control conditions (Dark), under moderate light at 200 *μ*mol photons m^−2^ s^−1^ (LL), or after 2 h of photoinhibitory illumination at 2,000 *μ*mol photons m^−2^ s^−1^ (HL). Experiments were repeated 4–6 times and representative images shown.

Singlet oxygen is a photosynthesis byproduct that is mainly formed at PSII under high-light conditions [36]. Because our data indicated that PsbS-KO and PsbS-RNAi rice has increased susceptibility to photoinhibition, we measured singlet oxygen production in wild type, PsbS-KO, and PsbS-RNAi leaves using singlet oxygen sensor green (SOSG) which specifically detects singlet oxygen [62,63]. Here, its fluorescence emission in wild-type plants increased almost four times by photoinhibitory illumination, and the increase of the SOSG fluorescence emission in PsbS-KO or PsbS-RNAi leaves was not significantly different from that in wild type (Figure 3a). We could get very similar results using dansyl-2, 2, 5, 5,-tetramethyl-2, 5-dihydro1*H*-pyrrole (DanePy) (data not shown).

Because high light-induced production of singlet oxygen was no higher in plants lacking PsbS than in the wild type, we measured the levels of other ROS, including superoxide and hydrogen peroxide. We visualized generation of the former by histochemically staining of rice leaves with nitroblue tetrazolium (NBT) (Figure 3b). In dark-adapted samples, no difference was observed among all genotypes, but both PsbS-KO and PsbS-RNAi leaves were stained dark-blue at 2,000 μmol photons m^−2^ s^−1^. Even under moderate light intensity (200 μmol photons m^−2^ s^−1^), more superoxide was accumulated in both PsbS-KO and PsbS-RNAi leaves than in the wild type.

Superoxide is rapidly dismutated to more stable hydrogen peroxide by SOD [28]. Therefore, we measured hydrogen peroxide production in wild type, PsbS-KO and PsbS-RNAi leaves by histochemically staining with 3, 3′-diaminobenzidine (DAB) (Figure 3c). Under photoinhibitory illumination at 2,000 μmol photons m^−2^ s^−1^ for 2 h, more hydrogen peroxide was detected in both PsbS-KO and PsbS-RNAi leaves than in the wild type.

To confirm this result, we visualized the production of singlet oxygen, superoxide, and hydrogen peroxide at high resolution, using a confocal laser scanning microscope with DanePy, dihydroethidium (DHE), and 2′,7′-dichlorofluorescein diacetate (DCFDA), respectively (Additional file 1: Figures S5-7), and the results were virtually the same as those observed by histochemical staining.

### Superoxide production is the initial event

**Figure 4:**
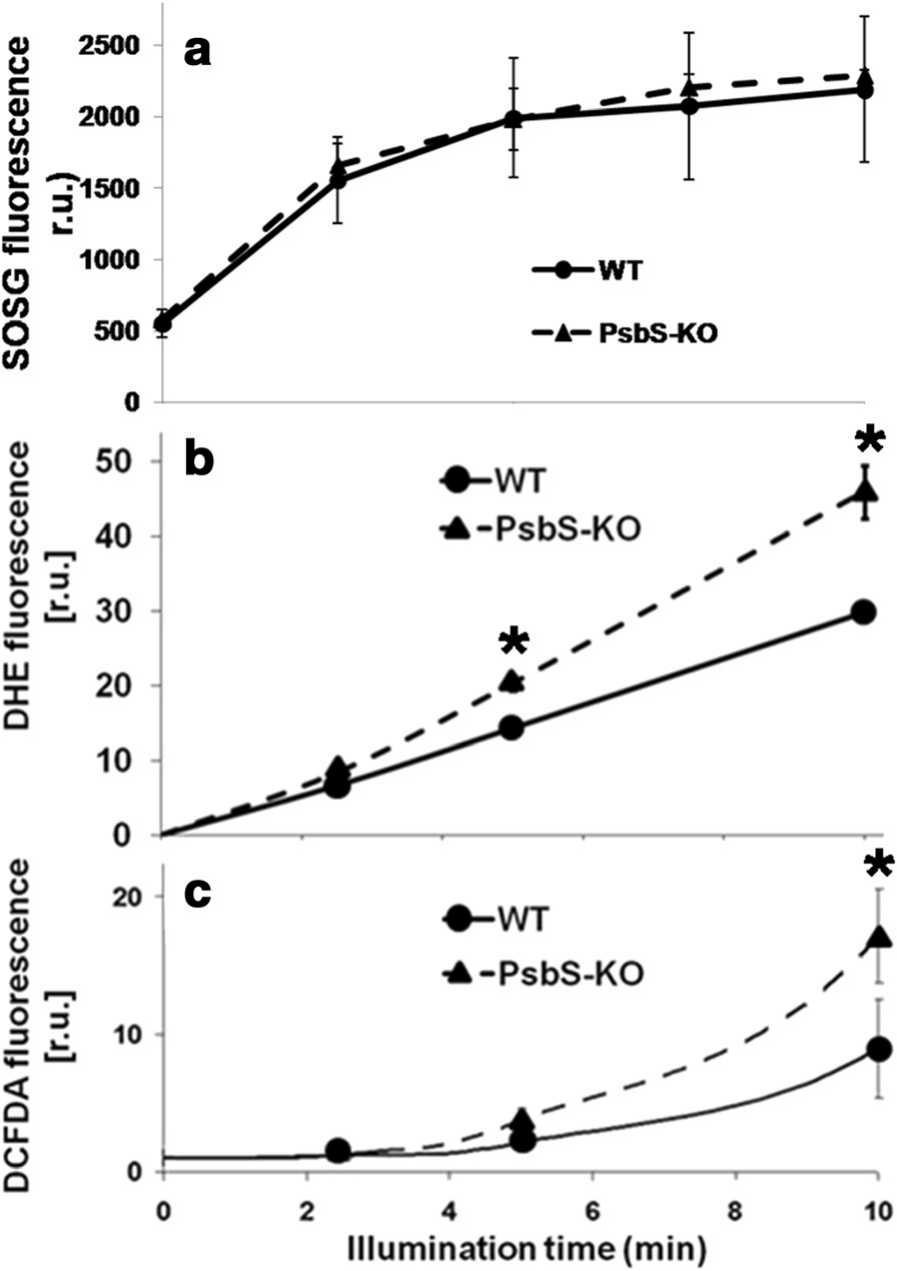
**Time course for generation of individual ROS in thylakoids of PsbS-KO and wild-type (WT) rice under photoinhibitory illumination at room temperature. (a)** Singlet oxygen production was monitored as relative increasing of SOSG (10 *μ*M) fluorescence at 530 nm. **(b)** Fluorescence emission of dihydroethidium (25 *μ*M) at 590 nm was used to detect superoxide production. **(c)** Fluorescence emission of DCFDA (10 *μ*M) at 525 nm was used to detect hydrogen peroxide. Thylakoid suspensions were illuminated at 700 *μ*mol photons m^−2^ s^−1^. Samples contained 10 *μ*g chlorophyll per mL. Each point represents mean of at least 4 experiments (SD indicated by bar; in some cases, SD is less than marker size) and the asterisks denote the results that were significantly different from those in the wild type (*P < 0.05). The statistical significance was evaluated using the Student’s *t* -test.

To confirm the results obtained using leaf segments, we then determined the levels of three ROS in isolated thylakoids before and after illumination with 700 μmol photons m^−2^ s^−1^ for 10 min. Although SOSG fluorescence emission increased by illumination, no significant differences in singlet oxygen generation were found between PsbS-KO and wild-type plants (Figure 4a). For more accurate detection of superoxide, we monitored increases in the fluorescence of DHE because it has been proven to detect superoxide in both intact cells and isolated subcellular fractions [64-66]. The suitability of DHE for assaying superoxide has also been verified by demonstrating that its fluorescence increases dose-dependently [64]. In the case of superoxide, the fluorescence emission at 615 nm rose linearly for 10 min, and the rate of increase was almost 40% as high in PsbS-KO thylakoids as in the wild type (Figure 4b). For hydrogen peroxide, the DCFDA fluorescence in thylakoids increased more rapidly in the PsbS-KO thylakoids (Figure 4c). However, production of hydrogen peroxide began 2 to 3 min after the start of superoxide generation. This suggested that most of the hydrogen peroxide resulted from the conversion of superoxide, thereby indicating that the main ROS overproduced in PsbS-KO plants is superoxide rather than hydrogen peroxide. In addition, the results obtained by using fluorescence sensors were confirmed by measuring the levels of superoxide and hydrogen peroxide based on NBT absorbance at 560 nm and DAB absorbance at 450 nm, respectively (Additional file 1: Figure S8).

### Superoxide produced at PSII is more in PsbS-KO rice leaves than in wild-type leaves

**Figure 5:**
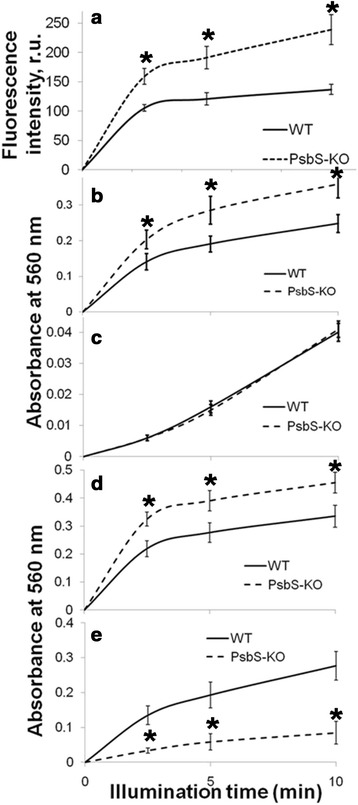
**Superoxide generated by photosystems of PsbS-KO and wild-type rice. (a)** PSII (BBY) particles. **(b)** Photosystem II complex isolated along sucrose gradient. **(c)** Photosystem I complex isolated along sucrose gradient. **(d)** Thylakoids with PSII-driven superoxide production. **(e)** Thylakoids with PSI-driven superoxide production. In **(a)**, fluorescence emission of dihydroethidium (25 *μ*M) at 590 nm was used to detect production. In **(b-e)**, absorbance of NBT (15 *μ*M) at 560 nm was used to detect production. Samples were illuminated at 700 *μ*mol photons m^−2^ s^−1^ for photoinhibition at room temperature. Each sample contained 10 *μ*g chlorophyll per mL. Each point represents mean of at least 4 experiments (SD indicated by bar) and the asterisks denote the results that were significantly different from those in the wild type (*P < 0.05). The statistical significance was evaluated using the Student’s *t* -test.

Under stress, superoxide is believed to be produced mostly in PSI [41,43]. In that case, PSI in PsbS-KO rice plants should be more damaged during photoinhibitory illumination where superoxide generated more than in wild-type plants. Upon photoinhibitory illumination, the decrease in P700^+^ formation in PsbS-KO was no greater than in the wild type [67], even though more superoxide was generated in the former. The increase in DHE fluorescence from PSII particles illuminated for 10 min was higher in PsbS-KO leaves (Figure 5a).

**Figure 6:**
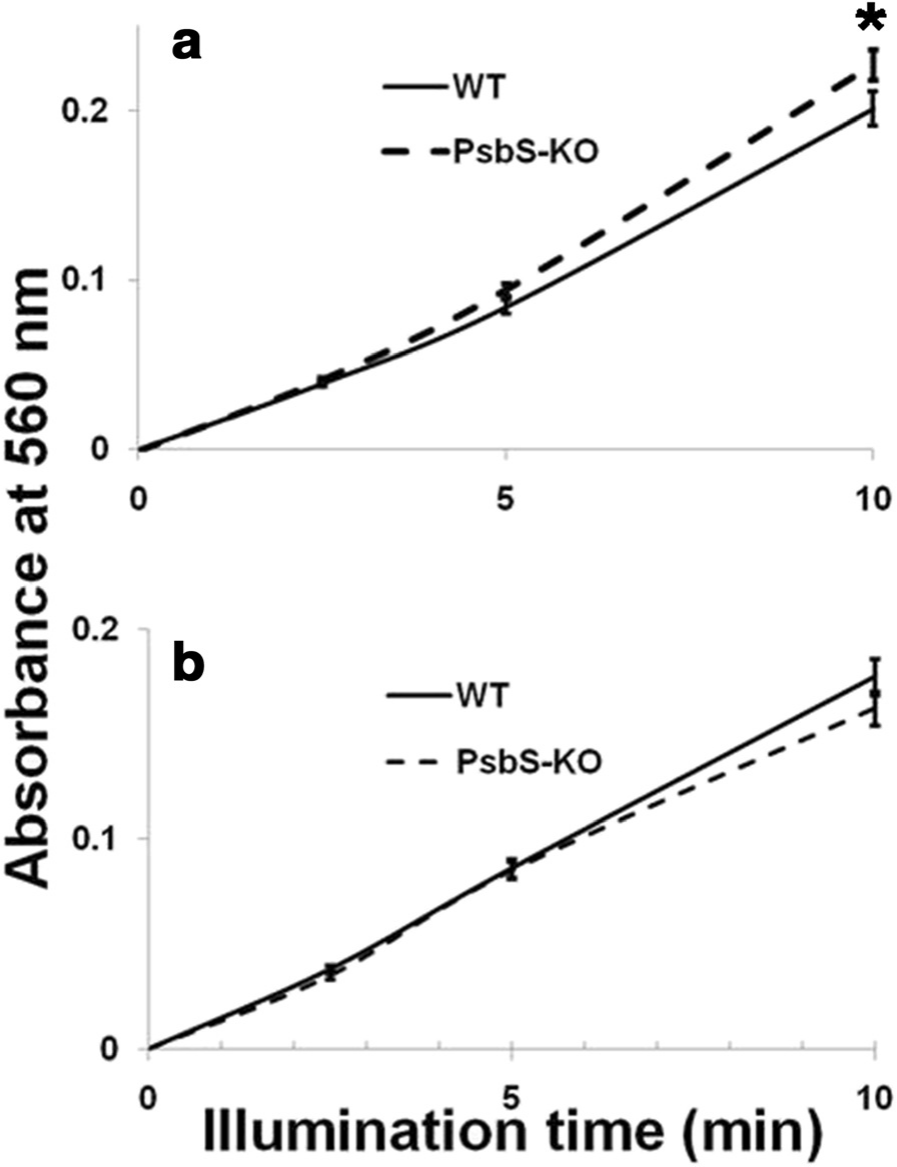
**Superoxide generated by whole electron transport chain and PSI of PsbS-KO and wild-type rice. (a)** Thylakoids with whole chain-driven production. **(b)** Thylakoids with PSI-driven production. NADP^+^ was used as final electron acceptor. Absorbance of NBT (15 *μ*M) at 560 nm was used to detect production. Samples were illuminated at 700 *μ*mol photons m^−2^ s^−1^ for photoinhibition at room temperature. Each sample contained 10 *μ*g chlorophyll per mL. Each point represents mean of at least 4 experiments (SD indicated by bar) and the asterisks denote the results that were significantly different from those in the wild type (*P < 0.05). The statistical significance was evaluated using the Student’s *t* -test.

These results were again confirmed by measuring changes in NBT absorbance at 560 nm, using PSI and PSII particles separated along a sucrose gradient [68]. As shown in Figures 5b,c, superoxide production in PSII particles was higher in PsbS-KO than in wild-type plants, whereas production in PSI complexes was similar for both genotypes. Because superoxide production was greater in PSII particles, we have compared protein composition of the PSI and PSII proteins using Western blotting (Additional file 1: Figure S9). Although BBY particles show contamination with PSI proteins, their amount do not differ significantly to affect superoxide production. We also measured PSI- and PSII-driven superoxide production in thylakoids using corresponding donor-acceptor pairs. As expected, PSII-driven production was higher in PsbS-KO (Figure 5d). Surprisingly, PSI-driven production was lower in PsbS-KO thylakoids (Figure 5e). These results were verified by measuring changes in NBT absorbance at 560 nm. When we instead used NADP^+^ as an electron acceptor, whole chain-driven superoxide production was again higher in PsbS-KO thylakoids (Figure 6a). However, PSI-driven production was lower in those thylakoids (Figure 6b). Presumably, this decrease was a consequence of the activation of cyclic electron flow around PSI in the PsbS-KO plants [67].

To make sure that the differences in superoxide production are not due the differences in electron transport rates, we measured electron transport rates in all samples by a Clark-type electrode (Table 1). As expected, we observed no striking differences in rates between wild-type and PsbS-KO thylakoids. Moreover, isolated PSI and PSII samples showed similar rates. Despite the differences noted in Fv/Fm between wild type and PsbS-KO leaves after photoinhibitory illumination for 2 h (Figure 2a), we could not observe such differences between the electron transport rates of two plants during illumination (Additional file 1: Figure S3). In fact, we could also not observe such differences between the two plants during illumination of isolated thylakoids with 700 μmol photons m^−2^ s^−1^ for 10 min. In both thylakoids, the decrease in Fv/Fm was 40% of the initial value. Taken together, our data suggested that, in the absence of qE, excess energy is released to molecular oxygen via an electron transport reaction. The observed phenotype in PsbS-KO leaves was probably a consequence of increased superoxide generation in PSII.

**Table 1:** **Photosynthetic electron transport rate (ETR) of rice thylakoids and isolated photosystems**

**Sample**	**Whole chain ETR**	**PSII-driven ETR**	**PSI-driven ETR**	**ETR of isolated PSII**	**ETR of isolated PSI**
WT	158 ± 12	275 ± 8	215 ± 45	334 ± 35	185 ± 65
PsbS-KO	147 ± 14	269 ± 10	245 ± 55	341 ± 18	180 ± 70

PSII-driven ETR and ETR of isolated PSII were measured by oxygen evolution (H_2_O to phenyl-p-benzoquinone), and whole chain ETR (H_2_O to methyl viologen), PSI-driven ETR and ETR of isolated PSI (sodium ascorbate and 2,6-dichlorophenol-indophenol to methyl viologen) were measured by oxygen consumption. Unit: ***μ***mol O_2_ (mg Chl)^−1^ h^−1^.

**Figure 7:**
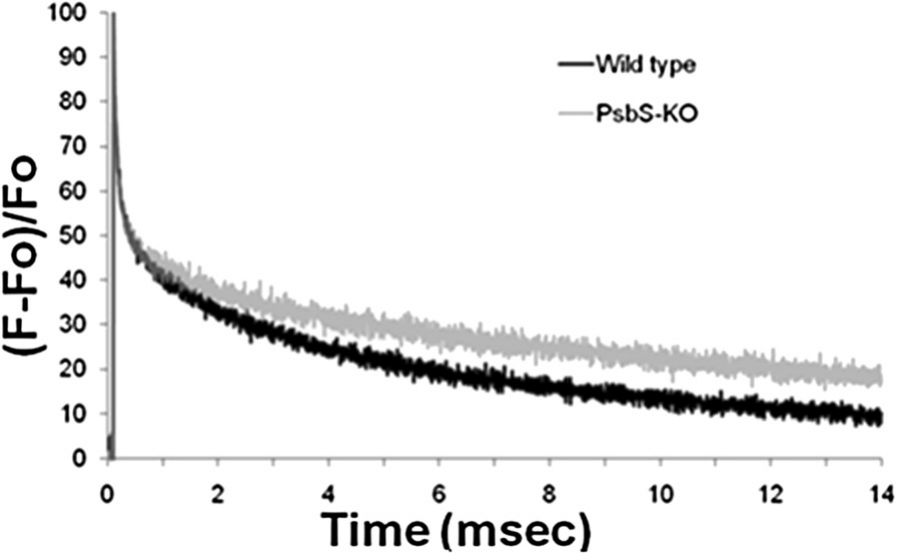
**Chlorophyll fluorescence decay after a single turnover flash in wild-type and PsbS-KO thylakoids**. For each experiment at least 12–15 repetitive flashes were given every 15 s; 24 traces from two independent experiments were averaged and plotted after normalization to the maximum fluorescence yield in the averaged trace.

Superoxide can be produced at different sites within PSII, such as through cyclic electron flow with the participation of cytochrome b_559_ [46] or at the Q_A_ site [45]. Therefore, we measured the redox state of cytochrome b_559_ in Mn-depleted PSII complexes as well as the re-oxidation of Q_A_^−^ in wild-type and PsbS-KO leaves. No significant differences between genotypes were found in the redox difference spectra for the high-potential form of cytochrome b_559_ in Mn-depleted PSII complexes (Additional file 1: Figure S10). However, we observed a difference in Q_A_^−^ re-oxidation kinetics, measured as Chl fluorescence decay, after a single turnover flash in the wild-type and PsbS-KO thylakoids (Figure 7, Additional file 1: Table S1).

## Discussion

In this study, we used biophysical, biochemical, physiological, and molecular biological approaches to characterize rice plants lacking the PsbS protein at PSII. Our objective was to elucidate the role of PsbS in the photoprotective mechanism of the qE component of NPQ. We confirmed previous conclusions that PsbS-deficient plants lack energy-dependent quenching [57,69]. Furthermore, we demonstrated that i) under high-light stress, PsbS-deficient plants produce more superoxide, followed by greater generation of hydrogen peroxide but not singlet oxygen; ii) their PSII (but not PSI) centers are more sensitive to photooxidative stress under constant illumination; and iii) more superoxide is produced by PSII in PsbS-KO plants compared with the wild type, probably occurring at the Q_A_ site. Because the functions of PsbS are likely to be similar in rice and *Arabidopsis*, we believe that our data offer new insights into the role of PsbS and the qE type of NPQ.

Although qE is a major photoprotective mechanism of the photosynthetic apparatus in higher plants; its absence can affect the photochemical efficiency of PSII. However, this effect seems to be negligible under constant light conditions in rice, as is true in *Arabidopsis* [6]. Nevertheless, under fluctuating light, PsbS-deficient rice plants show growth retardation at the seedling stage (Additional file 1: Figure S4) and reduced fitness at the reproductive stage [57], which is similar to that reported with *Arabidopsis* [13]. These reductions are likely caused by an increase in oxidative stress in plants lacking PsbS, even though the ROS species that may underlie this effect have remained unknown.

The triple knock-out mutant of the moss, *Physcomitrella patens*, (*psbs lhcsr1 lhcsr2*) lacking NPQ has a far higher triplet chlorophyll steady-state level than wild type [70] suggesting that the level of the singlet oxygen also should be higher in the absence of NPQ. However, in the early stage of photoinhibition, when singly reduced Q_A_ is reversibly stabilized, the triplet chlorophyll is rapidly quenched by the interaction with Q_A_^−^, preventing formation of harmful singlet oxygen [71]. We assume that our experimental condition is similar to this case. In our experiment, in the early stage of photoinhibition we do not expect more damage to the mutants (Figures 2a,b) and consequently, our result is acceptable showing that the level of singlet oxygen was not significantly more in the PsbS mutant lines compared with wild type.

Singlet oxygen, which has been implied to inhibit D1 protein synthesis [72-74], does not accumulate in plants lacking qE. In contrast, hydrogen peroxide, which also can influence the D1 repair system [72,75], damages cells under photoinhibitory illumination and may cause oxidative bursts leading to cell death. Here, hydrogen peroxide as well as superoxide produced more in rice that lacked qE. To determine accurate levels of ROS in leaf tissues both in vivo and in vitro, one should apply a variety of methods and take multiple measurements [76,77]. Here, we used assay systems based on several fluorescent dyes and other ROS sensors. In the case of superoxide, all data obtained using NBT were confirmed using DHE because NBT acts as an electron acceptor for PSII ([78], Krieger-Liszkay, Cedex, France; (unpublished data)). From our perspective, it was acceptable to employ NBT for in vitro assays because electron transfer to NBT will be minimal in the presence of artificial electron acceptors. Because superoxide can be converted to hydrogen peroxide even without SOD enzymes, we might explain the increase in hydrogen peroxide by an initial rise in the production of superoxide. Although we cannot rule out the possibility that synthesis of hydrogen peroxide is also elevated in rice leaves lacking qE, our data (most importantly those from time-course experiments performed on isolated thylakoids) are much better explained if superoxide production is the initial event that eventually leads to the formation of hydrogen peroxide. Similar data were reported in a parallel study with *Arabidopsis* [79]. There, fluorescence sensors were used in the thylakoids, and EPR spin-trapping with 4-pyridyl-1-oxide-N-tert-butylnitrone was tested in intact leaves, in order to detect the hydroxyl radicals that are produced from hydrogen peroxide/superoxide. Furthermore, in the presence of 20 μM nigericin (which eliminates the proton gradient over the thylakoid and, hence, qE), the signals were similar between the wild type and *npq4*, indicating that increased ROS production was due to a lack of qE.

The main site of superoxide generation in thylakoids under high-light conditions is thought to be PSI [43,80]. However, several researchers have also demonstrated light-induced generation of superoxide in PSII [44-46]. Instead, we showed that, when PsbS-KO and wild-type plants were analyzed, leaves from the former produced more superoxide. In our comparison between the two plant sources, we found that more superoxide was produced only in PSII, not in PSI. Moreover, we found that PSI does not seem to incur more photodamage in treated plants, and we also observed superoxide production in PSII particles. In fact, the proof of the Mehler reaction mainly referred with data from algae, and it remains much more controversial for higher plants because of the deficiency of proof of the Mehler reaction in higher plants. Badger et al. [81] reviewed a number of studies with higher plants, algae and cyanobacteria that have attempted to quantify O_2_ fluxes under various conditions and their contributions to the energy dissipation. The authors conclude that the Mehler reaction is unlikely to support a significant flow of electron transport in C_3_ and Crassulacean acid metabolism plants (probably less than 10%). Thus they questioned the Mehler reaction as a significant source of ROS in higher plants [81]. This uncertainty was fully justified by Driever and Baker [82] who could not detect any evidence for significant light driven Mehler reaction at ambient CO_2_ levels in two plant species. In addition, the data presented in this study on the lack of increase in ROS production from PSI centres would be consistent with this view that not much Mehler reaction occurs in higher plants in vivo. Cytochrome b_559_ may also be involved in superoxide generation in PSII, based on reports that superoxide can be detected by EPR spectroscopic analysis of PSII particles isolated from a cytochrome b_559_ mutant of tobacco [46]. The detection of superoxide in isolated thylakoids by a voltammetric method suggests that when the photosynthetic electron transport chain becomes over-reduced, superoxide may be generated at the Q_A_ site of PSII [45]. Altering the Q_A_^−^ re-oxidation kinetics (Figure 7, Additional file 1: Table S1) suggests that the Q_A_ site of PSII, rather than cyclic electron flow involving cytochrome b_559_, is the superoxide generation site in PSII. Another reason for the involvement of Q_A_ in PSII in higher superoxide production in PsbS mutant is probably the shift of redox-potential of Q_A_ to a more negative value due to the lack of PsbS, similar to the A249S mutant of *Thermosynechococcus elongatus* [83], which can make Q_A_^−^ to be able to reduce the molecular oxygen. Because in normal conditions the redox-potential of Q_A_/Q_A_^−^ is −80 mV [84] while the redox-potential of O_2_/superoxide is −160 mV [41]. Because PsbS controls the conformation and organization of PSII supercomplexes [22-25], it is reasonable to predict more superoxide production at PSII in PsbS-KO leaves than in the wild type. It is likely that superoxide production at PSII has received little attention because it is so rapidly converted to hydrogen peroxide and because PsbS-dependent light dissipation provides such an efficient system of protection. In PsbS-deficient plants this protection, which may be an important component of the role of qE in vivo, is compromised, facilitating detection of superoxide release.

## Conclusions

This study demonstrate that the PsbS-deficient rice plants to compensate for their lack of qE appear to develop other mechanisms for releasing excess energy to molecular oxygen; those protective systems may be initially triggered by superoxide production in PSII. Studies in *Arabidopsis* have shown that these systems may also provide broader protection against other sources of stress. When grown in the field, plants lacking PsbS induce a metabolic and transcriptomic shift that activates defense response pathways [85], resulting in an increase in resistance against biotic stress [79,85]. Whether this response influences the susceptibility of rice mutants lacking PsbS, as it does in *Arabidopsis*, remains to be established. It is an open question whether one can enhance crop yields by manipulating NPQ levels [86]. However, the intricate interplay between costs and benefits for NPQ has apparently not resulted in the selection of plants with “maximal” levels of NPQ. Potential positive effects of photooxidative processes [85] may be one factor favoring plants with less capacity for NPQ and could help to achieve food security for the growing human population using less available resources [87]. We hope that future studies will provide a deeper understanding of why regulation of photosynthetic light harvesting has been so elegantly organized.

## Methods

### Plants and growth conditions

One-month-old wild-type and PsbS-KO mutant seedlings of rice (*Oryza sativa* L.) were grown in rice soil (pH 4.5-5.5; Nonghyup, SamhwaGreentech, Seoul, Republic of Korea) in a greenhouse under natural sunlight. Growth conditions included a 16-h photoperiod and temperatures of 28/22 ± 2°C (day/night). For some experiments, rice seeds were germinated and cultured for one week on a solid agar Murashige and Skoog nutrient medium (Duchefa Biochemie, The Netherlands). Unless otherwise stated, plants were dark-adapted for at least 4 h before measurements were taken.

### Isolation of OsPsbS-KO transgenic rice

Putative PsbS-knockout mutant plants were selected from T-DNA insertional knockout mutant lines. These were generated by transformation with a T-DNA vector (pGA2707) containing the promoter-less *GUS* gene next to the LB of the T-DNA [52]. Seeds segregating in the T2 generation or their amplified progenies were used for experiments. T-DNA flanking sequences were determined as previously described [53].

Genomic DNA was isolated from the leaves of three-week-old plants by the CTAB method [88]. Leaf samples were extracted with an Rtech® MM301 Mixer Mill (Rtech GmbH and Co., Germany). For genotyping, 2 μg of total DNA was used in PCR reactions with genomic DNA-specific primers: for *OsPsbS1*, 5′- ATCACCGGGAAGGGAATC –3′ (left) and 5′- GTCGTCGCTGACGAA –3′ (right); and for *OsPsbS2*, 5′-AGCGTGAAGAGGATGAAGA–3′ (left) and 5′-CCAAGAGAGCAAGCCAAGAT–3′ (right). A T-DNA-specific primer set was also used: 5′-TTGGGGTTTCTACAGGAC–3′ and 5′-AGAAGATCAAGGTGGGGACG–3′. PCR conditions for amplification were an initial 95°C for 5 min, followed by 30 cycles of 95°C for 1 min, 60°C for 1 min, and 72°C for 1 min; plus a final extension at 72°C for 10 min. The products were electrophoretically separated on a 0.8% (w/v) agarose gel containing ethidium bromide. DNA bands were visualized with an imaging system (Vilber Lourmat, France).

### Generation of OsPsbS RNAi transgenic rice

To generate an RNA interference vector for OsPsbS1, we amplified a 102-bp gene fragment by PCR, using forward primer 5′-ATAGGATCCCTCGAGCGCGCGGTGTCCGTCAAGAC-3′ and reverse primer 5′-GCGGAATTCAAGCTTGTCCTCGGTCTTGAACTTTG-3′. Afterward, the fragment was cloned into the XhoI-HindIII and BamHI-EcoRI sites of pFGL727 (pBSIIKS-Intron). A SacI-KpnI fragment of pBSIIKS-Intron-OsPsbS1 was transferred into the SacI-KpnI sites of pGA1611. The OsPsbS RNAi plasmid was then transformed into rice using *Agrobacterium* strain LBA4404 as previously described [89].

### Analysis of transcript levels and immunoblots

Total RNA was isolated as described in [90]. The gene-specific primers for *OsPsbS1* were amplified using forward primer 5′-CTGTTCGGCAGGTCCAAGAC-3′ and reverse primer 5′-TTCAGCTGCGCCAGGATTC-3′. PCR products were separated by electrophoresis on a 1.2% agarose gel. Immunoblots were conducted as previously described [91].

### Measurements of fluorescence and electron transport

After dark-adaptation for 10 min (in addition to the 4 hours), Chl fluorescence was measured at room temperature from detached leaves with a PAM2000 pulse-amplitude-modulated fluorometer (Walz, Effeltrich, Germany) in a room where influences from air-conditioning system are negligible. Actinic light was provided by a halogen lamp (Schott KL1500, Mainz, Germany). Fluorescence parameters Fm and Fm’ were induced by a saturating pulse of white light (0.8 s, 5,000 μmol photons m^−2^ s^−1^). Fm and Fo are defined as the maximal and minimal fluorescence yields of a “dark-adapted” sample when all PSII reaction centers are fully closed or opened, respectively. Fm' is the maximal fluorescence yield attained with a pulse of saturating light while leaves are illuminated by actinic light. We used Fv/Fm [or (Fm – Fo)/Fm] to monitor the potential efficiency of PSII photochemistry. Parameters for photochemical quenching (qP) and NPQ were calculated by the equations described in [92]. Electron transport rates were computed as previously described as: ETR = (Δ*F*/*F*m’) x PAR x 0.5 × 0.84 [Δ*F* = (Fm’ - Ft)] assuming equal distribution of excitation between PSI and PSII [93]. For kinetic analysis of Q_A_^−^ re-oxidation, fluorescence decay in wild-type and PsbS-KO thylakoids was recorded after a single turnover flash [94]. The data were fitted to a three-component exponential decay equation after normalization.

The extent of P700 photooxidation, P700^+^, was assessed from differential changes in absorbance (810 nm minus 860 nm) using a PAM101/102/103 fluorometer (Walz, Germany) in the reflectance mode [95]. After 5 min of pre-illumination (120 μmol m^−2^ s^−1^, actinic white light), values for P700^+^ were calculated as (I_max_ – I_min_) ⁄ I_max_, where I_max_ is the signal intensity after applying saturating far-red light, and I_min_ is the signal intensity [93,96]. This pre-illumination was done to overcome any possible acceptor-side limitation of PSI that might have prevented full P700 oxidation.

### Photoinhibitory treatment

To inflict photodamage to PSII with and without lincomycin, in vivo photoinhibition was induced by illuminating leaf segments with 2,000 μmol photons m^−2^ s^−1^ of white light from metal halide lamps at 27°C of leaf surface. For lincomycin treatment, leaf segments were infiltrated with 2 mM lincomycin by submergence [97] for 12 h under darkness, and the leaf segments were kept floated on the same treatment solution during photoinhibitory illumination. For recovery, samples were kept under 50 μmol photons m^−2^ s^−1^ of white light from a fluorescence lamp to induce the repair of damaged PSII. Following photoinhibitory illumination, the tissues were dark-adapted for 30 min prior to fluorescence measurements, unless otherwise stated.

### Histochemical staining of superoxide and hydrogen peroxide

Histochemical staining for ROS production was conducted as previously described [98-100], with some modifications. For superoxide determinations, leaf samples were immersed in 6 mM NBT solution containing 50 mM sodium phosphate (pH 7.5) for 12 h in the dark. To detect hydrogen peroxide, detached leaves from wild-type and mutant plants were immersed in 5 mM DAB solution containing 10 mM MES (pH 3.8) for 12 h under darkness. Both reactions were stopped by soaking the leaves with lacto-glycerol-ethanol (1:1:4 by vol) and boiling in water for 5 min. The cleared leaves were preserved in 50% ethanol and photographed.

### Determination of ROS levels in isolated thylakoids and photosystems by fluorescence emission analysis and absorbance of ROS sensors

The fluorescence emission spectra of the ROS sensors were acquired with an F-4500 fluorescence spectrophotometer (Hitachi, Japan). Singlet oxygen was detected both in leaves and in thylakoids as described in [62,63]. To detect superoxide, we examined the fluorescence of dihydroethidium according to the method described in [64]. Fluorescence of DCFDA was used to detect hydrogen peroxide [101]. Leaf segments from one-month-old seedlings were submerged for 12 to 14 h (25°C under darkness) in a fluorescent sensor solution for infiltration.

The absorbance of each ROS sensor was measured by an UV-1650PC UV-Visible spectrophotometer (Shimadzu, Japan). To detect superoxide, NBT absorbance was measured at 560 nm as described in [102]. The absorbance of DAB at 450 nm was used to detect hydrogen peroxide [103].

Superoxide production by PSI- and/or PSII-driven electron transport in the thylakoids was monitored with corresponding donor-acceptor pairs. To detect levels of individual ROS generated by PSII-driven electron transport, we used a reaction buffer containing 0.1 M sucrose, 10 mM NaCl, 10 mM KCl, 5 mM MgCl_2_, 10 mM Tricine, 1 mM KH_2_PO_4_, and 0.2% BSA (pH 8.0). The following ingredients were added to this buffer immediately prior to the experiments: 30 mM sodium ascorbate to mediate de-epoxidation of violaxanthin to zeaxanthin; 3 mM phenyl-p-benzoquinone to mediate PSII-driven electron transport; and 0.3 mM ATP to fuel ATP hydrolysis. To detect levels of individual ROS generated by PSI-driven electron transport, we again used the buffer described above. The following ingredients were added immediately before those experiments began: 5 mM NH_4_Cl as an uncoupler of the oxygen-evolving complex; 20 μM 3-(4,4-dichlorophenyl)-1,1-dimethylurea (DCMU) as a PSII electron transport inhibitor; 30 mM sodium ascorbate and 0.1 mM 2,6-dichlorophenol-indophenol as an electron donor for PSI; 50 μM methyl viologen only or 1 mM NADP^+^ supplemented with 10 μM ferredoxin to mediate linear electron transport; and 0.3 mM ATP to fuel ATP hydrolysis. ROS sensors were also added: 15 μM NBT to detect superoxide and 15 μM DAB for hydrogen peroxide. In all experiments the Chl concentration was 10 μg mL^−1^. Samples were illuminated at 700 μmol photons m^−2^ s^−1^ for photoinhibition.

### Isolation of thylakoids and PSI and PSII complexes and detection of cytochrome b_559_

Thylakoids were isolated from PsbS-KO and wild-type plants according to the method described in [104]. PSII complexes (BBY particles) were prepared by Triton X-100 purification, as described in [105]. Those particles were stored at −80°C in a re-suspension medium containing 400 mM sucrose, 15 mM NaCl, 5 mM MgCl_2_, and 40 mM Mes (pH 6.5). To confirm that there are no large differences in protein composition of thylakoids and BBY particles (except PsbS protein) in wild-type and PsbS-KO plants, we have conducted SDS-PAGE with Coomassie staining (Additional file 1: Figure S11), and the result showed that there are no large differences in the protein compositions between wild-type and PsbS-KO plants. Light-induced redox changes in the spectra of high-potential cytochrome b_559_ were determined as described in [106]. Mn-depleted PSII complexes were prepared by incubating the BBY particles in 0.8 M Tris (pH 8.0) for 20 min [46]. Afterward, the isolated PSII complexes were washed several times in a re-suspension medium and stored at −80°C. The PSII and PSI complexes were also isolated by sucrose-gradient centrifugation after solubilizing the thylakoids through the addition of octyl glucoside (0.88% (w/v)) and sodium dodecyl sulfate (0.22% (w/v)) [68]. Superoxide production was monitored in those isolated complexes by using corresponding donor-acceptor pairs for PSI and PSII. The concentration of Chl was determined in 80% acetone extracts as described in [107].

## Additional file

Additional file 1:
**Figure S1.** Genotyping of the PsbS-KO mutant line. **Figure S2.** Light-response curves for NPQ. **Figure S3.** Light-response curves for electron transport rates. **Figure S4.** Relative growth rates of 1-week-old seedlings. **Figure S5.** Production of singlet oxygen. **Figure S6.** Detection of superoxide anion radical production. **Figure S7.** Detection of hydrogen peroxide production. **Figure S8.** Time course for ROS production in thylakoids. **Figure S9.** Immunoblotting of thylakoids and BBY particles. **Figure S10.** Light-induced redox changes in high-potential cyt b_559_. **Figure S11.** Protein composition of thylakoids and BBY particles. **Table S1.** Analysis of decay time constant (τ_i_, μs) and amplitude (A_i_, arbitrary units) of Q_A_^−^ reoxidation kinetics after a single-turnover flash.
