# Supplementary material for: Production of superoxide from Photosystem II in a rice (Oryza sativa L.) mutant lacking PsbS
Source: BMC Plant Biol. 2014 Oct 24;14:242. doi: 10.1186/s12870-014-0242-2 (PMC4219129; doi:10.1186/s12870-014-0242-2)
Supplement: Additional file 1: — Figure S1. Genotyping of the PsbS-KO mutant line. Figure S2. Light-response curves for NPQ. Figure S3. Light-response curves for electron transport rates. Figure S4. Relative growth rates of 1-week-old seedlings. Figure S5. Production of singlet oxygen. Figure S6. Detection of superoxide anion radical production. Figure S7. Detection of hydrogen peroxide production. Figure S8. Time course for ROS production in thylakoids. Figure S9. Immunoblotting of thylakoids and BBY particles. Figure S10. Light-induced redox changes in high-potential cyt b559. Figure S11. Protein composition of thylakoids and BBY particles. Table S1. Analysis of decay time constant (τi, μs) and amplitude (Ai, arbitrary units) of QA − reoxidation kinetics after a single-turnover flash. [file 12870_2014_242_MOESM1_ESM.pdf]

Additional file

Production of superoxide from Photosystem II in a rice (*Oryza sativa* L.) mutant lacking PsbS

Ismayil S. Zulfugarov[1,5,6], Altanzaya Tovuu[1,7], Young-Jae Eu[1], Bolormaa Dogsom[1], Roshan Sharma Poudyal[1], Krishna Nath[1], Michael Hall[2], Mainak Banerjee[4], Ung Chan Yoon[4], Yong-Hwan Moon[1], Gynheung An[3], Stefan Jansson[2] and Choon-Hwan Lee[1*]

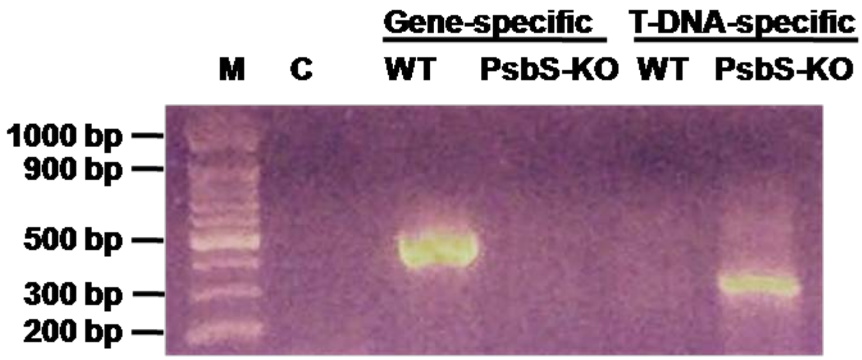

Figure S1. Genotyping of the PsbS-KO mutant line.

Genotyping was done as described in Materials and Methods. M, molecular weight markers; C, water control; WT, wild type; PsbS-KO, PsbS knockout line; Gene-specific, gene-specific primers; T-DNA-specific, T-DNA specific primers. The expected product size for the gene-specific primers set is 459 bp; for the T-DNA-specific primers set is 280bp.

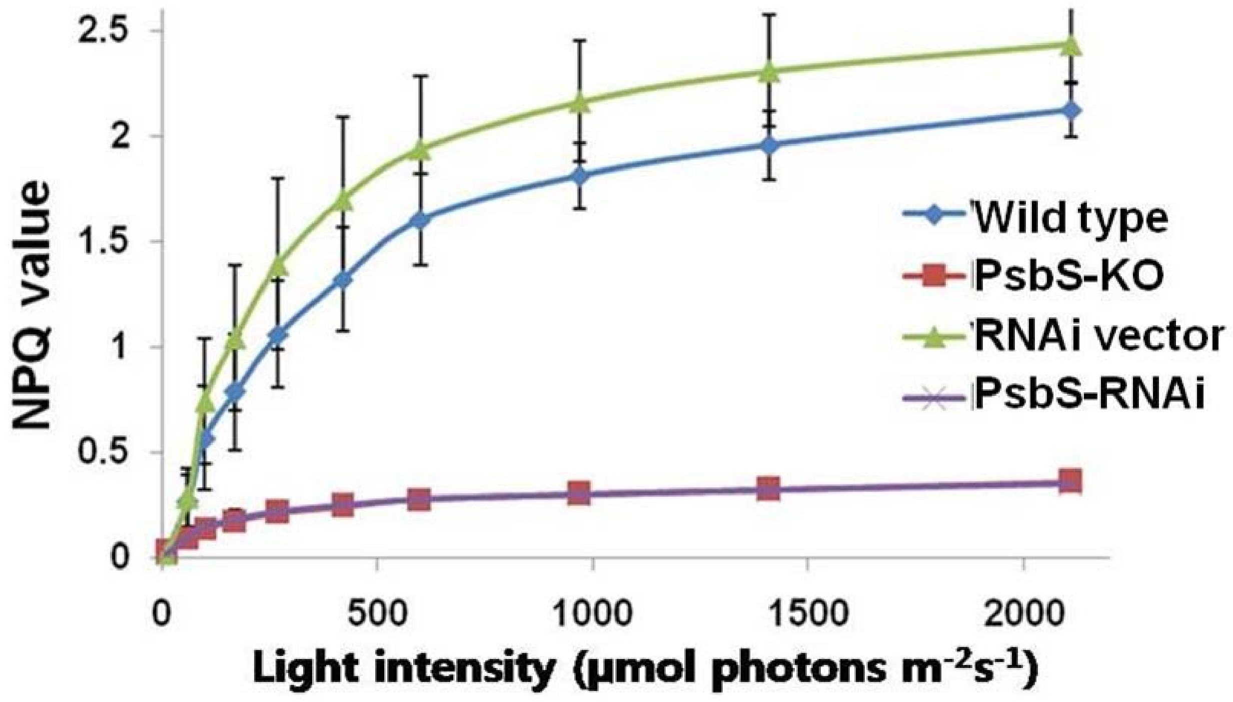

Figure S2. Light response curves for NPQ.

Leaves of 1-month-old seedlings grown in greenhouse were dark-adapted for 10 min before measurements. Photosynthetically active irradiance of 0, 70, 110, 180, 250, 400, 550, 900, 1,400, or 2,200 μmol photons m^{-2} s^{-1} was then applied for 10 min. Each point represents mean of at least 4 experiments (SD indicated by bar).

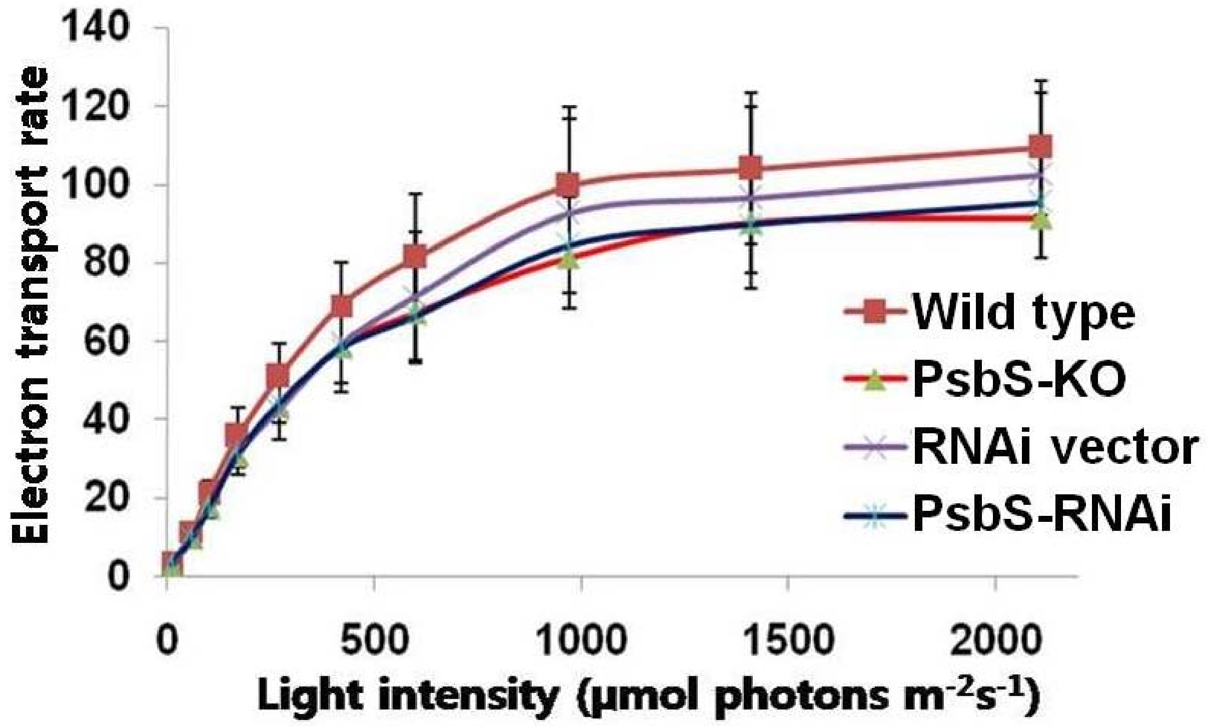

Figure S3. Light response curves for electron transport rates.

Leaves of 1-month-old seedlings grown in greenhouse were dark-adapted for 10 min before measurements. Photosynthetically active irradiance of 0, 70, 110, 180, 250, 400, 550, 900, 1,400, or 2,200 μmol photons m^{-2} s^{-1} was then applied for 10 min. Each point represents mean of at least 4 experiments (SD indicated by bar).

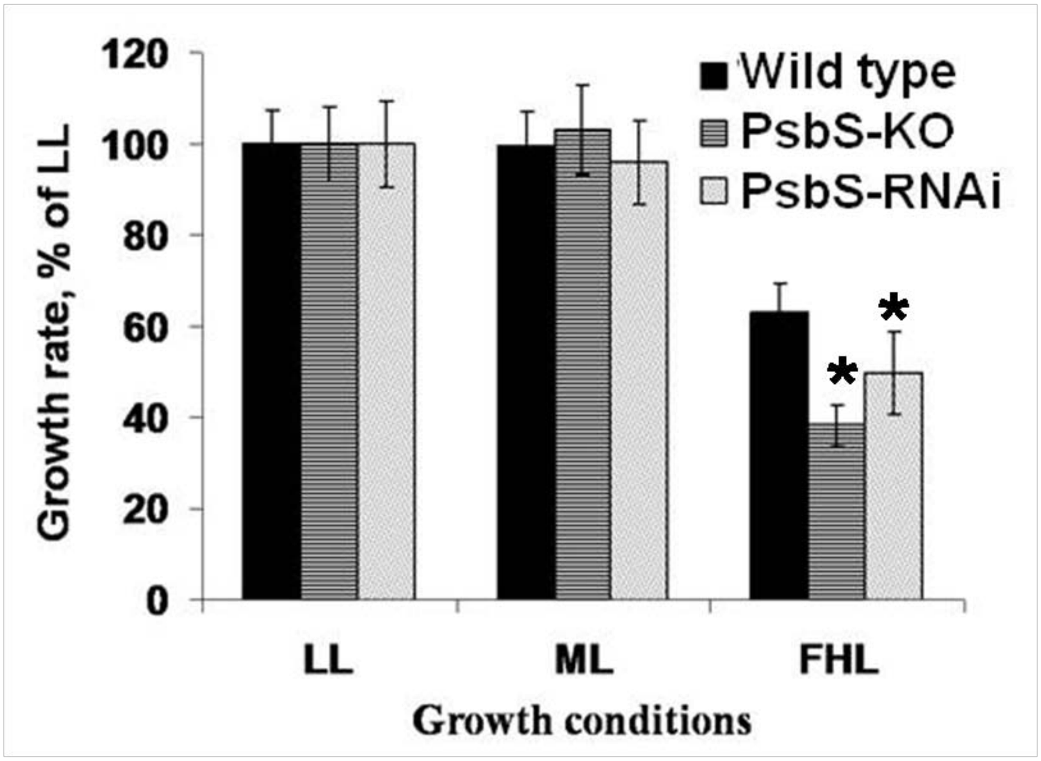

Figure S4. Relative growth rates of 1-week-old seedlings.

Plants were grown on solid (agar) Murashige and Skoog media under different light conditions; a low-light (LL), 30 µmol photons $m^{-2} s^{-1}$; a medium-light (ML), 300 µmol photons $m^{-2} s^{-1}$; a natural fluctuating light in a greenhouse (FHL), 50 to 1,500 µmol photons $m^{-2} s^{-1}$. All plants were grown on agar plate at the same growth conditions in the culture room except the light intensity; for FHL which grown in greenhouse under fluctuating light. To measure growth rate we have used plants heights only. Each point represents mean of at least 4 experiments (SD indicated by bar) and the asterisks denote the results that were significantly different from those in the wild type (*$P < 0.05$). The statistical significance was evaluated using the t-test.

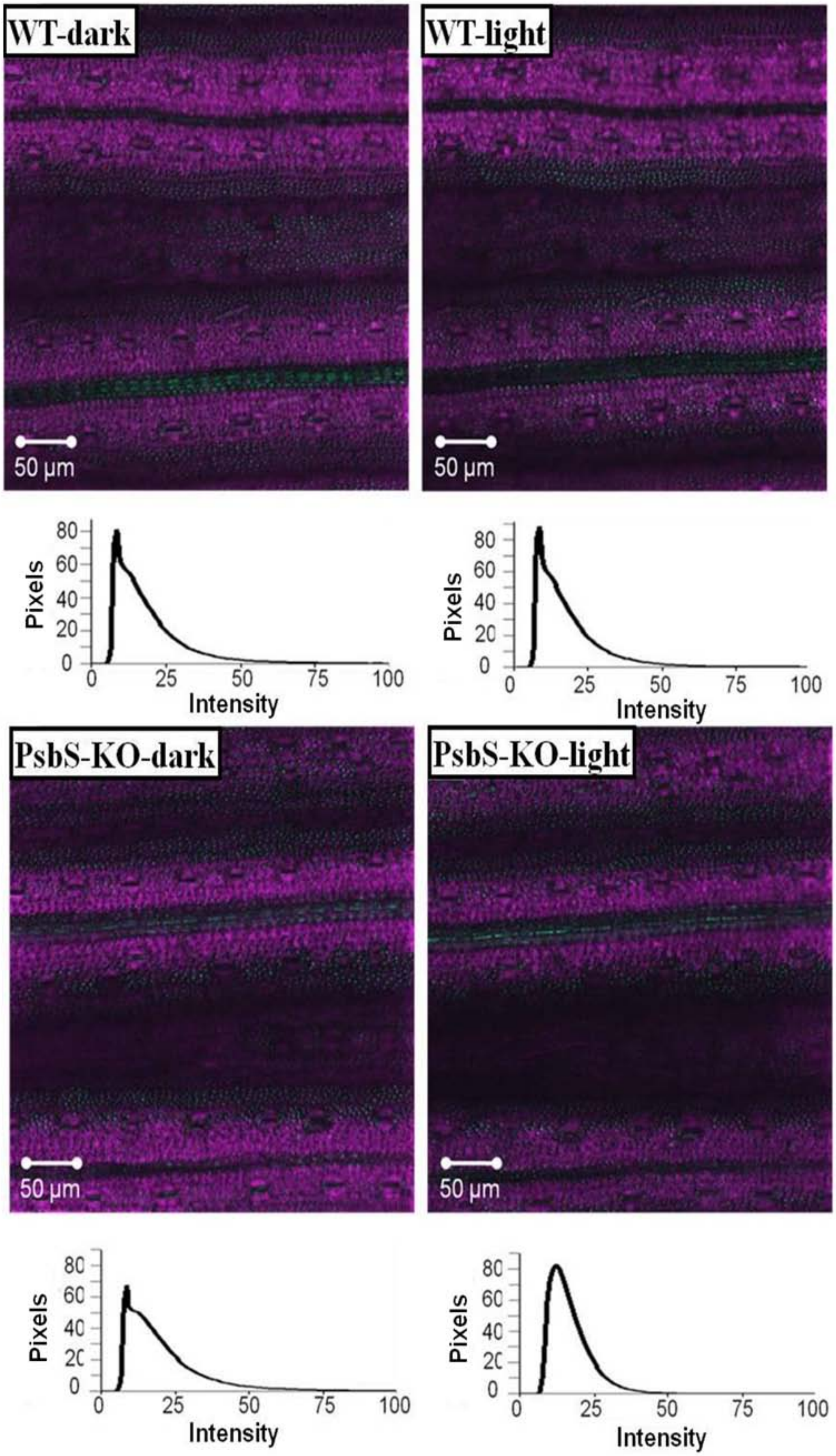
WT-dark
WT-light
50 μm
Pixels
Intensity
0
20
40
60
80
0
25
50
75
100
PsbS-KO-dark
PsbS-KO-light

Figure S5. Production of singlet oxygen.

Root systems of 1-week-old seedlings were infiltrated with DanePy (2 mM) for 1 h under darkness. Merged confocal fluorescence images of leaves from wild type (WT) and PsbS-KO taken before (dark) and after (light) exposure to photoinhibitory illumination (1,000 μmol photons m^{-2} s^{-1} for 10 min at room temperature). Distribution plot for intensities of all 1024 x 1024 pixels is shown beneath each digitized confocal image. Similar results were obtained from 6 independent experiments.

To detect singlet oxygen in samples of our wild type and mutant plants we used the fluorescent sensor DanePy (Hideg et al., 2002), synthesized as described by Kàlai et al. (2002). We tested singlet oxygen production in isolated thylakoids to check the quality of our synthesized DanePy; singlet oxygen production in isolated thylakoids was carried out as described in Fufezan et al. (2002) in the presence of two types of herbicide (DCMU and bromoxynil) (data not shown). The fluorescence from DanePy and Chl was then monitored using a Zeiss (Oberkochen, Germany) LSM510 confocal laser scanning microscope with Kr/Ar laser excitation. Leaves of one-week-old rice seedlings were imaged after infiltration with DanePy with a 25X objective by using 364 nm laser excitation. Channel mode detection was used to record the emissions of DanePy (505 to 550 nm). A single channel was used throughout an entire experiment and corrected for autofluorescence and Chl fluorescence by collecting a 488-nm excitable signal before photoinhibitory treatment. Image acquisition conditions were kept constant for comparison between control and illuminated leaves. Confocal images were analyzed using LSM 510 Image Examiner software. Whole confocal images were used to make the pixel distribution plots, and the distribution of dark pixels was ignored. The selected experimental conditions, including excitation UV-laser beam intensity and emission detection threshold, were as described by Hideg et al. (2006) to minimize autofluorescence from the leaves.

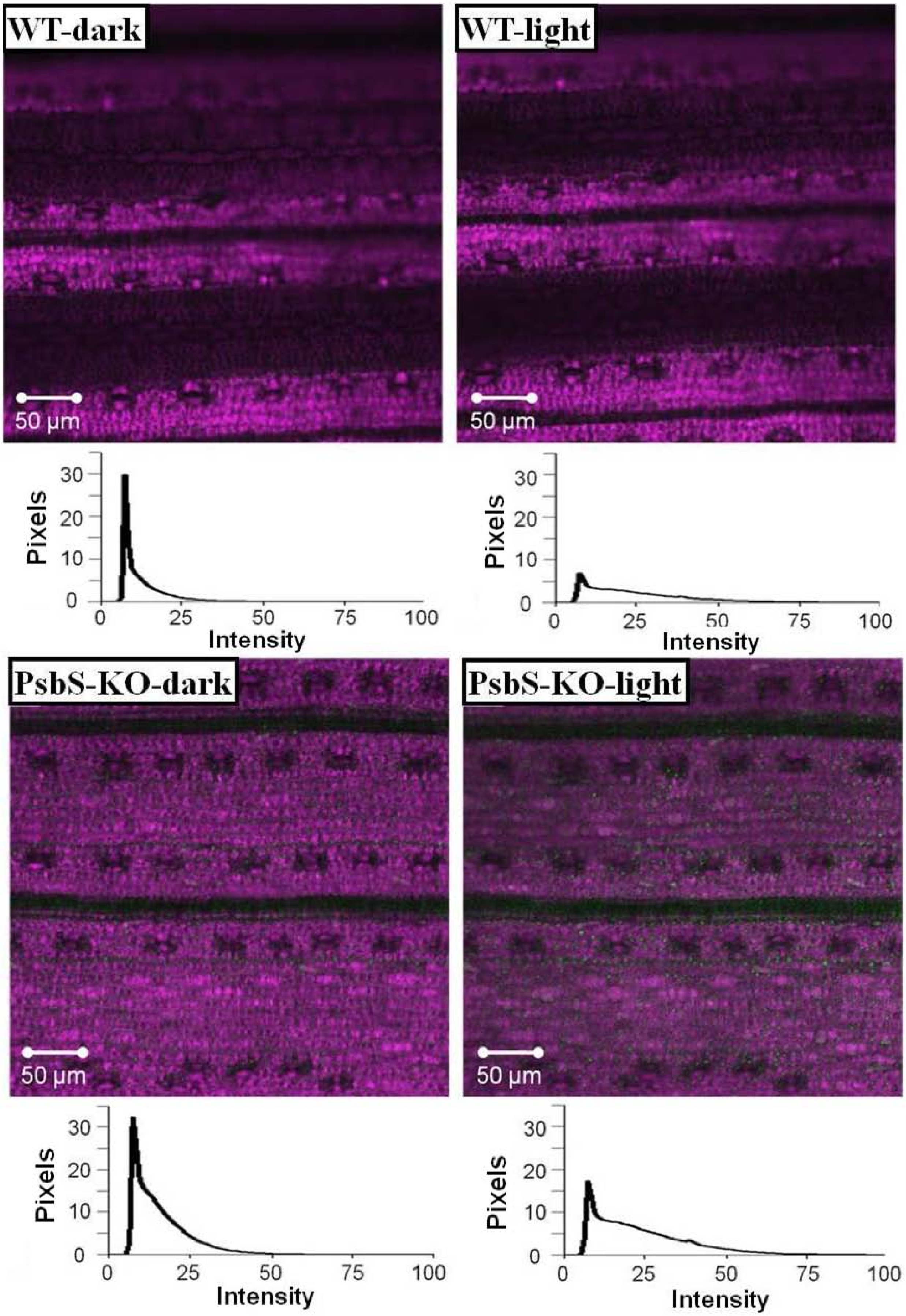
WT-dark
50 μm
Pixels
Intensity
WT-light
50 μm
Pixels
Intensity
PsbS-KO-dark
50 μm
Pixels
Intensity
PsbS-KO-light
50 μm
Pixels
Intensity

Figure S6. Detection of superoxide anion radical production.

Leaves of wild-type (WT) and PsbS-KO seedlings were infiltrated with dihydroethidium (25 µM) under darkness. Merged confocal fluorescence images were taken before (dark) and after (light) exposure to photoinhibitory illumination (1,000 µmol photons m^{-2} s^{-1} for 10 min at room temperature). Distribution plot for intensities of all 1024 x 1024 pixels is shown below each digitized image. Similar results were obtained from 6 independent experiments. Pixel distribution plots showed that after 10 min of photoinhibitory illumination in both wild type and PsbS-KO rice leaves, the frequencies of the low intensity pixels decreased with subsequent increases in the frequencies of the high intensity pixels, and this shift was more apparent in PsbS-KO rice leaves. Similar results were obtained in six independent experiments. Channel mode detection was used to record the emissions of DHE (550 to 650 nm). Another experimental conditions are the same as in Figure S5 legend.

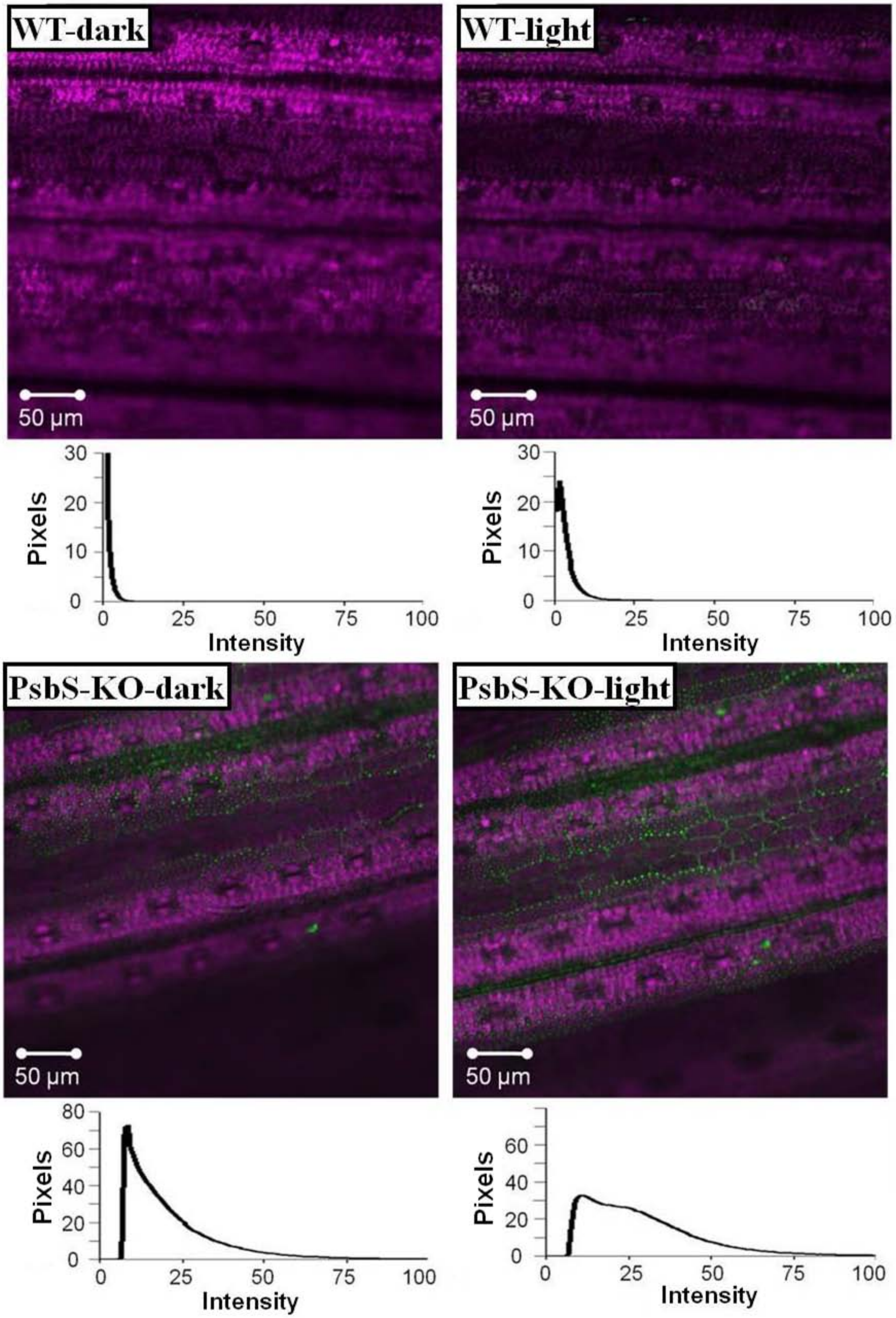
WT-dark
50 µm
Pixels
Intensity
WT-light
50 µm
Pixels
Intensity
PsbS-KO-dark
50 µm
Pixels
Intensity
PsbS-KO-light
50 µm
Pixels
Intensity

Figure S7. Detection of hydrogen peroxide production.

Leaves from wild-type (WT) and PsbS-KO seedlings were infiltrated with 2',7'-dichlorofluorescein diacetate (DCFDA) (10 µM) under darkness. Merged confocal fluorescence images were taken before (dark) and after (light) exposure to photoinhibitory illumination (1,000 µmol photons $m^{-2}s^{-1}$ for 10 min at room temperature). Distribution plot for intensities of all 1024 x 1024 pixels is shown beneath each digitized image. Similar results were obtained from 6 independent experiments. Pixel distribution plots indicated that DCFDA fluorescence increased more in PsbS-KO leaves than in wild-type leaves after photoinhibitory illumination for 10 min. Similar results were obtained in six independent experiments. Channel mode detection was used to record the emissions of DCFDA (505 to 550 nm). Another experimental conditions are the same as in Figure S5 legend.

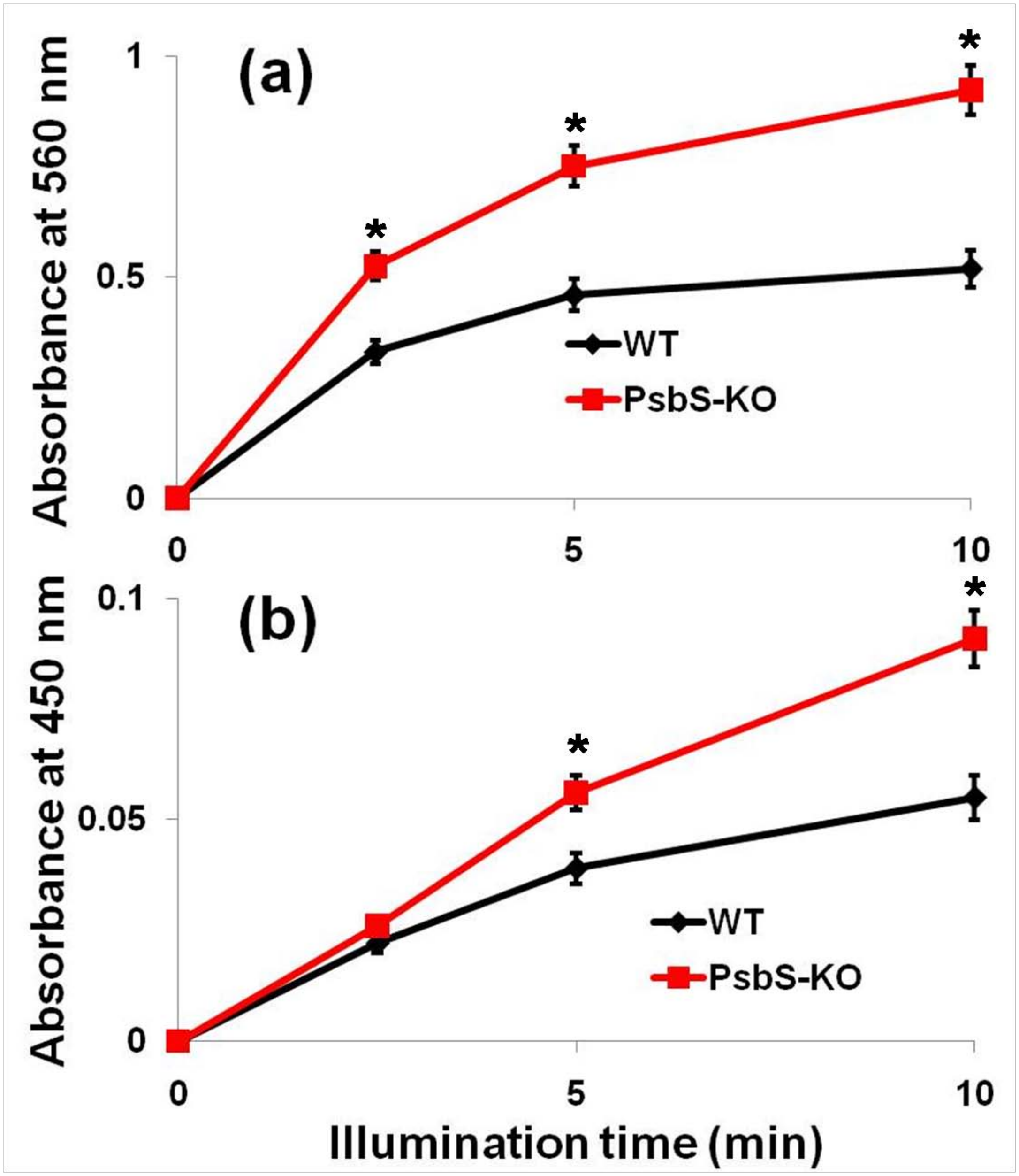

Figure S8. Time course for ROS production in thylakoids.

(a) Superoxide production monitored as increase in NBT absorbance (15 µM) at 560 nm.

(b) Hydrogen peroxide production monitored as increase in DAB absorbance (15 µM) at 450 nm. Thylakoid suspensions were illuminated at 700 µmol photons m^{-2} sec^{-1} for photoinhibition at room temperature. Each sample contained 10 µg of Chl per mL. +SOD, superoxide dismutase (5 units per mL) added to reaction mixture. Each point represents mean of at least 3 experiments (SD indicated by bar).

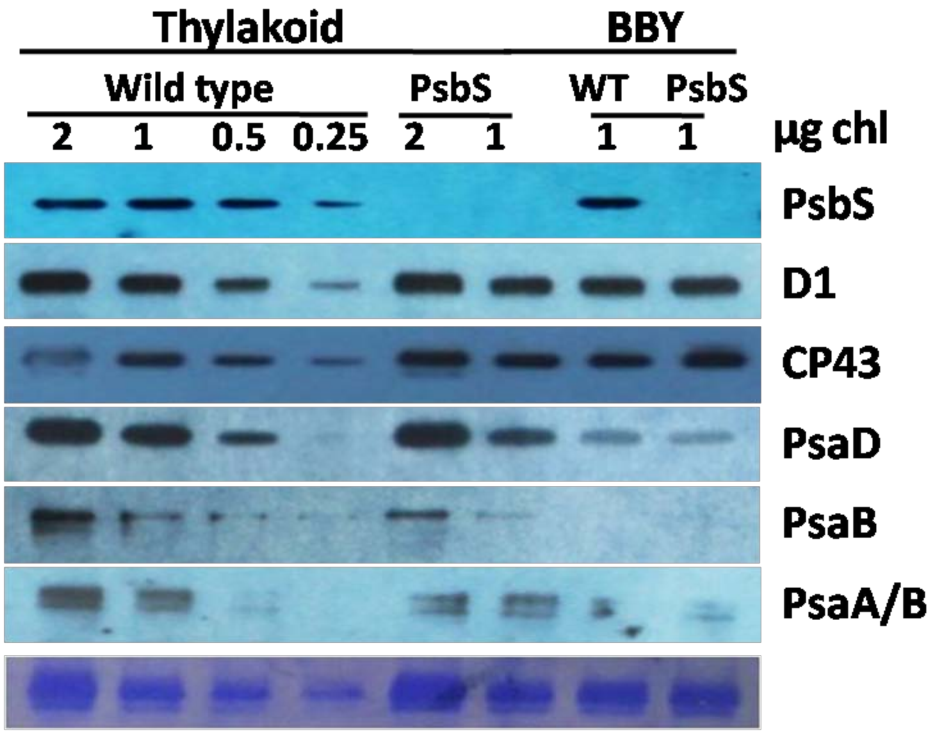

Figure S9. Immunoblotting of thylakoids and BBY particles.

The thylakoids and BBY particles of the wild-type and PsbS-KO mutant plants were subjected to immunoblot using different antibodies against PSI and PSII proteins. Bottom line is the coomassie staining of the samples shows major LHCII for loading control.

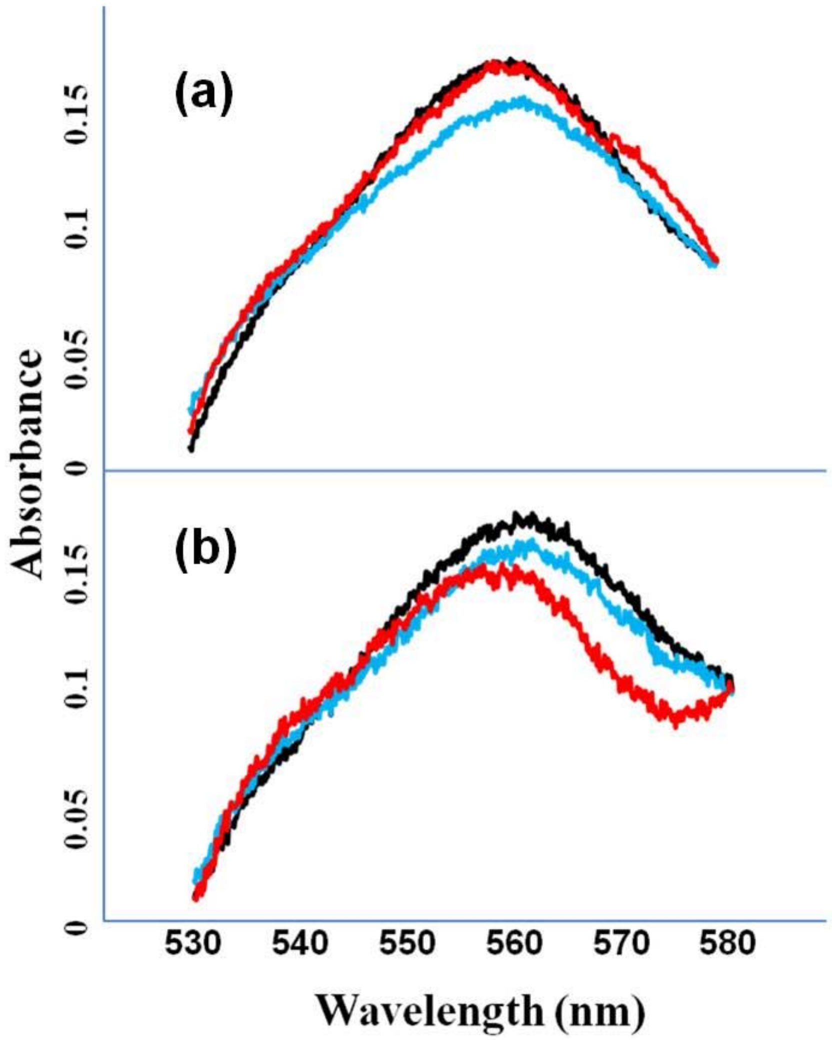

Figure S10. Light-induced redox changes in high-potential cyt b_{559}.

(a) Wild type.

(b) PsbS-KO.

Redox difference spectra measured in Mn-depleted (Tris-treated) PSII complexes under darkness and after illumination. Samples (100 μg Chl mL^{-1}) were illuminated with white light (1,000 μmol photons m^{-2} s^{-1}). Dark lines − hydroquinone minus ferricyanide spectra; Blue lines – 100-s illumination minus ferricyanide-oxidized spectra; Red lines – 300-s illumination minus ferricyanide-oxidized spectra.

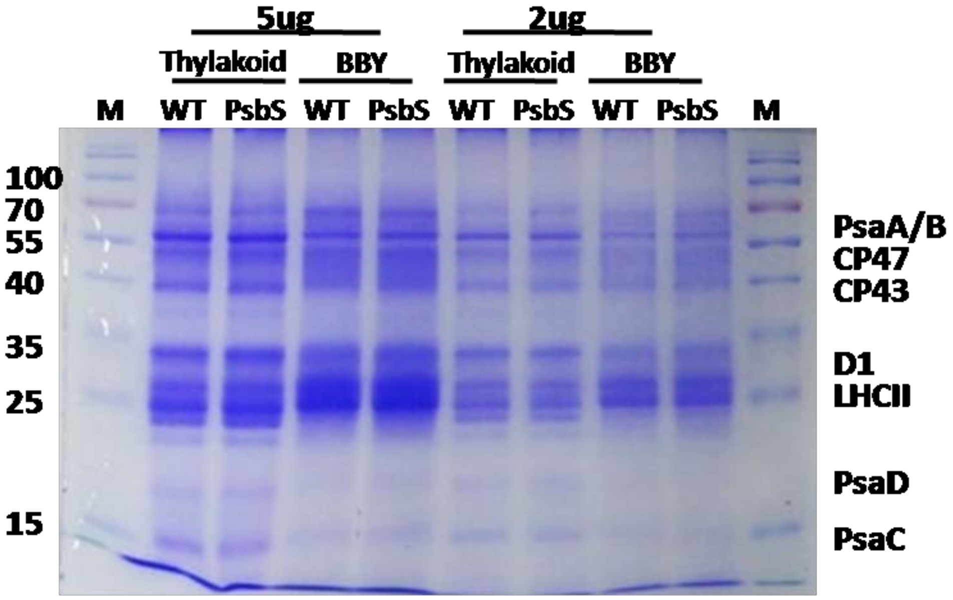

Figure S11. Protein composition of thylakoids and BBY particles.

The thylakoids and BBY particles of the wild-type and PsbS-KO mutant plants were subjected to SDS-PAGE and stained with coomassie. M, molecular weight markers; WT, wild type; PsbS, PsbS-KO line.

Table S1. Analysis of decay time constant (τ_i, μs) and amplitude (A_i, arbitrary units) of Q_A^- reoxidation kinetics after a single-turnover flash. Traces shown in Figure 8 were used for the analysis.

Sample	τ_1	A_1	τ_2	A_2	τ_3	A_3
Wild type	0.037±0.002	39.09±1.12	0.193±0.007	28.13±1.10	3.131±0.013	31.13±0.12
PsbS-KO	0.049±0.002	49.57± 0.83	0.321±0.013	20.58±0.76	4.256±0.023	28.09±0.13

The data were fitted to a three-component exponential decay equation after normalization.
